# A fiber-deprived diet causes cognitive impairment and hippocampal microglia-mediated synaptic loss through the gut microbiota and metabolites

**DOI:** 10.1186/s40168-021-01172-0

**Published:** 2021-11-11

**Authors:** Hongli Shi, Xing Ge, Xi Ma, Mingxuan Zheng, Xiaoying Cui, Wei Pan, Peng Zheng, Xiaoying Yang, Peng Zhang, Minmin Hu, Tao Hu, Renxian Tang, Kuiyang Zheng, Xu-Feng Huang, Yinghua Yu

**Affiliations:** 1 Jiangsu Key Laboratory of Immunity and Metabolism, Department of Pathogen Biology and Immunology, Xuzhou Medical University, Xuzhou, 221004 Jiangsu China; 2 Illawarra Health and Medical Research Institute (IHMRI) and School of Medicine, University of Wollongong, Wollongong, NSW 2522 Australia; 3 State Key Laboratory of Animal Nutrition, College of Animal Science and Technology, China Agricultural University, Beijing, 100193 China; 4 Queensland Brain Institute, The University of Queensland, St. Lucia, QLD 4113 Australia

**Keywords:** Dietary fiber deficiency, Cognition, Gut microbiota, Short-chain fatty acids, Gut-brain axis

## Abstract

**Background:**

Cognitive impairment, an increasing mental health issue, is a core feature of the aging brain and neurodegenerative diseases. Industrialized nations especially, have experienced a marked decrease in dietary fiber intake, but the potential mechanism linking low fiber intake and cognitive impairment is poorly understood. Emerging research reported that the diversity of gut microbiota in Western populations is significantly reduced. However, it is unknown whether a fiber-deficient diet (which alters gut microbiota) could impair cognition and brain functional elements through the gut-brain axis.

**Results:**

In this study, a mouse model of long-term (15 weeks) dietary fiber deficiency (FD) was used to mimic a sustained low fiber intake in humans. We found that FD mice showed impaired cognition, including deficits in object location memory, temporal order memory, and the ability to perform daily living activities. The hippocampal synaptic ultrastructure was damaged in FD mice, characterized by widened synaptic clefts and thinned postsynaptic densities. A hippocampal proteomic analysis further identified a deficit of CaMKIId and its associated synaptic proteins (including GAP43 and SV2C) in the FD mice, along with neuroinflammation and microglial engulfment of synapses. The FD mice also exhibited gut microbiota dysbiosis (decreased Bacteroidetes and increased Proteobacteria), which was significantly associated with the cognitive deficits. Of note, a rapid differentiating microbiota change was observed in the mice with a short-term FD diet (7 days) before cognitive impairment, highlighting a possible causal impact of the gut microbiota profile on cognitive outcomes. Moreover, the FD diet compromised the intestinal barrier and reduced short-chain fatty acid (SCFA) production. We exploit these findings for SCFA receptor knockout mice and oral SCFA supplementation that verified SCFA playing a critical role linking the altered gut microbiota and cognitive impairment.

**Conclusions:**

This study, for the first time, reports that a fiber-deprived diet leads to cognitive impairment through altering the gut microbiota-hippocampal axis, which is pathologically distinct from normal brain aging. These findings alert the adverse impact of dietary fiber deficiency on brain function, and highlight an increase in fiber intake as a nutritional strategy to reduce the risk of developing diet-associated cognitive decline and neurodegenerative diseases.

Video Abstract

**Supplementary Information:**

The online version contains supplementary material available at 10.1186/s40168-021-01172-0.

## Background

Neurodegenerative diseases, including Alzheimer’s disease (AD, the most prevalent form of dementia), have devastating impacts on the individual affected, their families, and health and social care systems [1]. Epidemiological evidence suggests that unhealthy diets and lifestyle factors can increase the risk of developing AD [2, 3]. The Lancet commission concluded that 40% of dementia cases could be prevented by addressing modifiable risk factors, including diet [1]. A global diet survey showed that the majority of adults have a severely deficient dietary fiber intake. For example, the average fiber intake per person is about 15 g/day in the USA [4], 13.6 g/day in the UK [5], and 11 g/day in China [6]. This is significantly lower than the 25–35 g/day of fiber intake, which is recommended by the World Health Organization (WHO). A strong link, however, is yet to be established between low dietary fiber intake and an increased risk of developing neurodegenerative diseases.

Dietary fiber consists of complex carbohydrates, which are neither digested nor absorbed but instead fermented in the gastrointestinal tract. The dietary fiber thus shapes the gut microbiota and impacts levels of fermentative end products, such as short-chain fatty acids (SCFAs) [7]. Recent evidence suggests that gut microbiota are able to modulate brain function and behaviors via the “microbiota-gut-brain” axis [8], but its mechanism of action remains poorly understood. For example, transplantation of microbiota isolated from donors on a high-fat diet into otherwise healthy mice disrupts the intestinal barrier and induces cognitive decline in those animals [9]. In germ-free mice or antibiotic-treated rodents, gut microbiota dysbiosis influences hippocampal neurogenesis and brain development through microglia activation [10, 11]. Extending these studies, we investigated how fiber deficiency-induced gut dyshomeostasis may contribute to cognitive decline via the gut-brain axis. Emerging research suggests an intriguing interaction between the gut, brain, and immune system, wherein any dysregulated communication could disrupt neuro-immune homeostasis and induce neuropathology. For example, intestinal permeability is increased in dementia patients with endotoxemia, a threefold increase in serum lipopolysaccharide (LPS, the major outer membrane component of Gram-negative bacteria) [12, 13]. It is known that LPS can activate microglia and trigger a neuroinflammatory response [14, 15], which has been linked to synapse loss and cognitive decline in both humans and animal studies [16]. However, no studies thus far have examined the impact of a fiber-deficient diet on the ultrastructure and proteomic profile of synapses, or on immune-microglial polarization within the brain region that regulates cognitive function.

Here, for the first time, we determined the effects of dietary fiber deficiency on cognition using a mouse model of chronic dietary fiber deficiency (FD), which mimics a sustained low fiber intake in humans. The synaptic ultrastructure, proteome profiles, and microglial-neuroinflammation-synapse pathway were examined in the hippocampus, as were measurements of gut homeostasis (including gut microbiota profiles, colonic mucosa, and the epithelium barrier, as well as serum SCFAs and LPS). Furthermore, to establish a causal association between fiber deficiency-induced gut dyshomeostasis and cognitive decline, the microbiota-gut-brain axis was also examined within a short-term version of the fiber deficiency mouse model. Finally, to assess a potential role of SCFAs (metabolites of fiber fermentation by gut microbiota) in mediating gut-brain dysfunction, the parameters of the gut-brain axis were examined in SCFA receptor knockout (GPR41^−/−^ and GPR43^−/−^) mice, and also in mice with SCFA supplementation in a fiber-deficient diet.

## Results

### Dietary fiber deficiency induces cognitive impairment and synaptic ultrastructure alteration

**Fig. 1:**
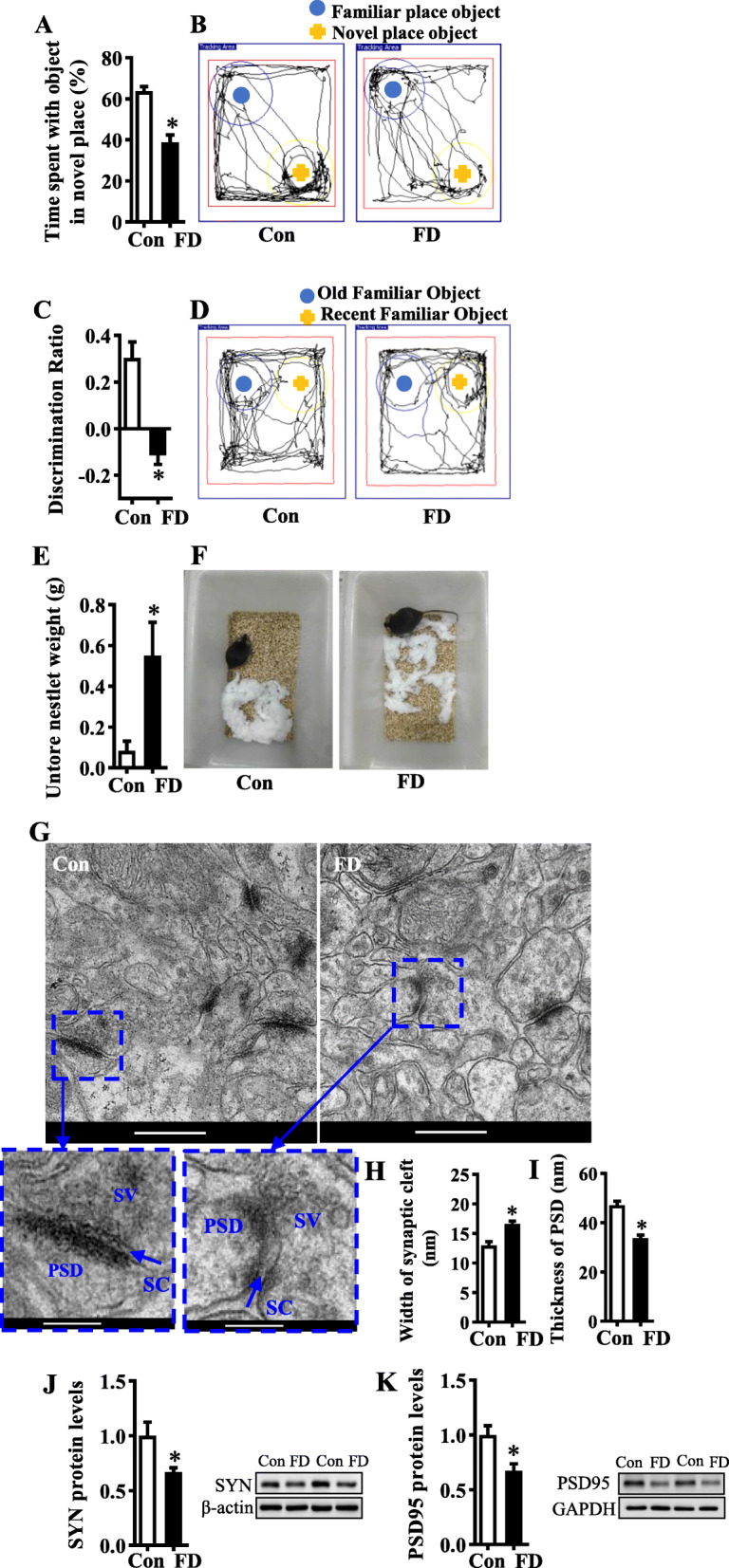
Dietary fiber deficiency for 15 weeks promoted cognitive impairment and alteration in synaptic ultrastructure. The object location, temporal order memory and nest-building tests were performed to evaluate mice’s cognition (**A**–**F**) (*n* = 15). **A**, **B** The object location test. **A** Percentage of time spent with the object in the novel place to total object exploration time. **B** Representative track plots of Con and FD groups recorded by SMART Video tracking system in the testing phase. Blue dot and yellow crucifix denote an object retained in the old place and moved to the novel place in the testing phase. **C**, **D** The temporal order memory test. **C** The discrimination ratio. **D** Representative track plots of Con and FD groups recorded by SMART Video tracking system in the testing phase. Blue dot and yellow crucifix denote an old familiar object and a recent familiar object in the testing phase, respectively. **E**, **F** The nest-building test was used to assess the activity of the daily living of mice. **E** Untore nestlet weight. **F** Representative nest of Con and FD groups. **G** The ultrastructure of synapses on the electron micrograph in the hippocampus CA1 region of mice fed with different diets (scale bar: 500 nm). The second row’s enlarged images were from the first row in the area indicated with a dotted line box (scale bar: 200 nm). **H**, **I** Image analysis of the width of the synaptic cleft and the thickness of PSD (*n* = 3). PSD: postsynaptic density; SC: synaptic cleft; SV: synaptic vesicle. **J**, **K** The protein level of SYN and PSD95 in the hippocampus (*n* = 6). Values are mean± SEM. *p < 0.05 fiber deficiency (FD) group vs. control (Con) group

After 15 weeks of the FD diet, mice showed significant cognitive deficits, including impaired object location memory, temporal order memory, and the ability to perform activities of daily living (Fig. 1A–F). Both the place discrimination index in the object location test and temporal order discrimination index in the temporal order memory test were significantly decreased in FD mice compared with the control mice (*p* < 0.05, Fig. 1A–D). The difference in object location and temporal order memory between the FD and control group was not due to the variability in general activity since the total exploration time of objects during the testing phase was comparable between the two groups (Figure S1A and B). The FD group displayed a decreased ability to build a nest in the nesting behavior test, as these mice had a higher untorn nestlet weight and lower deacon nest score than that of the controls (both *p* < 0.05, Fig. 1E, F and Figure S1C). Plasticity of synaptic ultrastructure in the hippocampus is required for cognition [17]. Transmission electron microscopy showed that the synaptic ultrastructure of the CA1 region of the hippocampus was altered in FD mice. The synaptic clefts were widened, and the postsynaptic densities were thinned in the FD mice (both *p* < 0.05, Fig. 1G–I). Furthermore, the presynaptic and postsynaptic markers, synaptophysin (SYN), and postsynaptic density protein 95 (PSD95), were reduced in the FD group (both *p* < 0.05, Fig. 1J, K). In addition, the energy intake, body weight and adipose tissues weight were higher in the FD group compared with the control group after 15 weeks (Figure S1D-F)**.**

### Dietary fiber deficiency induces the dysregulation of hippocampal CaMKIId and its associated synaptic proteins.

**Fig. 2:**
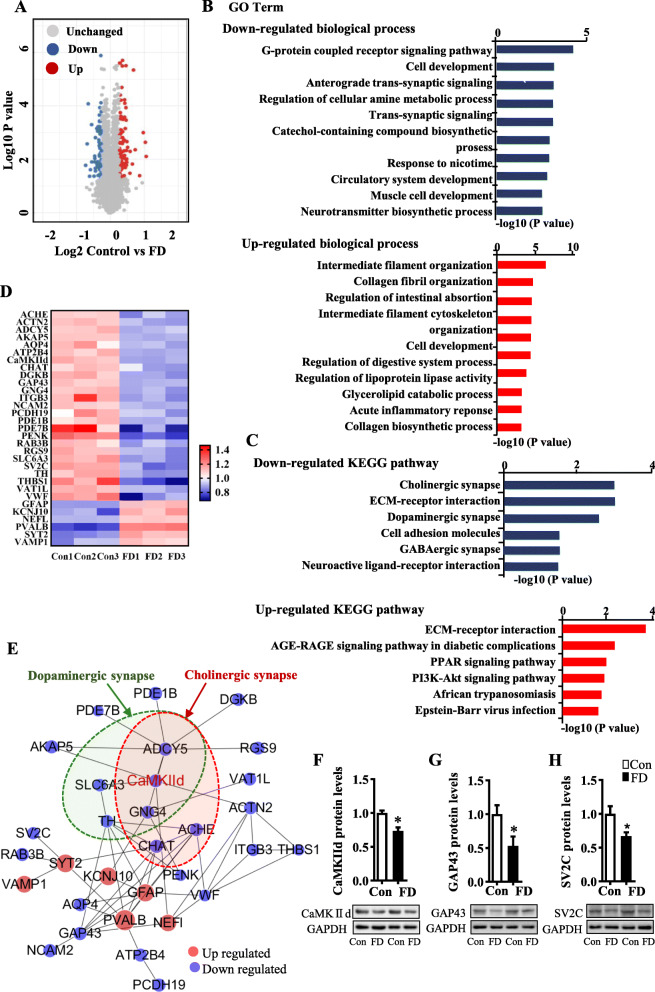
Dietary fiber deficiency for 15 weeks altered synaptic proteome in the hippocampus. **A**–**E** The quantitative analysis of the global proteome of the hippocampus (*n* = 3). **A** Volcano plot of differentially expressed proteins. **B** Analysis of GO enrichment of biological process for differentially expressed proteins. **C** Analysis of KEGG pathway of the differentially expressed synaptic proteins. **D** The heatmap of the differentially expressed synaptic proteins. **E** PPI network analysis of distinctly dysregulated proteins based on STRING database and Cytoscape 3.6.0. Black lines denoted the interaction between two proteins. The red node signified up-regulation, blue node signified downregulation. **F**–**H** The verification of the quantitative analysis of the global proteome of the hippocampus for downregulated proteins (CaMKIId, GAP43, and SV2C) by western blot (*n* = 5–6). Values are mean ± SEM. *p < 0.05 fiber deficiency (FD) group vs. control (Con) group

To identify hippocampal synaptic proteins involved in fiber deficiency-induced cognitive impairment, we compared the hippocampal proteomic profiling between the FD and the control mice using mass-spectrometry-based proteomics analysis. There were 68 downregulated proteins and 92 upregulated proteins in the FD group (Fig. 2A). The GO analysis was used to determine the top 10 biological processes relevant to these proteins (Fig. 2B). These include the G-protein coupled receptor signaling pathway, cell development, anterograde trans-synapse signaling, regulation of cellular amine metabolic process, and trans-synaptic signaling. The altered proteins were further analyzed using KEGG pathways and assigned into down-regulated synaptogenesis pathways, including cholinergic synapses, dopaminergic synapses, cell adhesion molecules, gamma-aminobutyric acid (GABAergic) synapses, neuroactive ligand-receptor interactions, and extracellular matrix (ECM)-receptor interactions (all *p* < 0.05, Fig. 2C). With respect to the upregulated pathways, KEGG pathway analysis showed ECM-receptor interaction, advanced glycation end product and their receptors (AGE-RAGE) signaling pathway in diabetics, the peroxisome proliferator-activated receptor (PPAR) signaling pathway, the phosphoinositide 3-kinases (PI3K)-Akt signaling pathway, African trypanosomiasis, and Epstein Barr-virus infection (all *p* < 0.05, Fig. 2C).

Thirty-one proteins within synaptogenesis pathways were differentially expressed in the hippocampus between the FD and the control mice (Fig. 2D). Altered synaptic proteins induced by the FD diet were mapped onto the protein-protein interactions (PPI) network (Fig. 2E). The PPI network indicated that calcium/calmodulin-dependent protein kinase II delta (CaMKIId or CaMK2d) is a core node between the cholinergic synapse and dopaminergic synapse pathways. CaMKIId directly interacted with adenylate cyclase 5 (ADCY5), solute carrier family 6 member 3 (SLC6A3), G protein subunit gamma 4 (GNG4), and choline acetyltransferase (CHAT) within the two synapse pathways as well as other synaptic proteins, such as A-kinase anchoring protein 5 (AKAP5) (a postsynaptic scaffold protein) and actin (ACTN)2 (which mediates spine morphology and assembly of the postsynaptic density). CaMKIId also interacted indirectly with some proteins involved in synaptic plasticity and cognitive function, such as growth-associated protein (GAP)43 (a crucial component of the axon and presynaptic terminals) and synaptic vesicle glycoprotein 2V (SV2C) (a membrane glycoprotein localized to synaptic vesicles). These two synaptic proteins are also reduced in dementia patients [18]. Next, the significant variants detected in the proteomic analysis were verified in the hippocampus using immunoblot. We found that the levels of CaMKIId, GAP43, and SV2C proteins were decreased in the hippocampus of FD groups compared to control animals (*p* < 0.05, Fig. 2F–H). Overall, the proteomics results suggest that the dysregulation of CaMKIId and its associated synaptic proteins contributes to fiber deficiency-induced synaptic and cognitive impairments.

### Dietary fiber deficiency leads to hippocampal microglia M1 polarization and synaptic engulfment

**Fig. 3:**
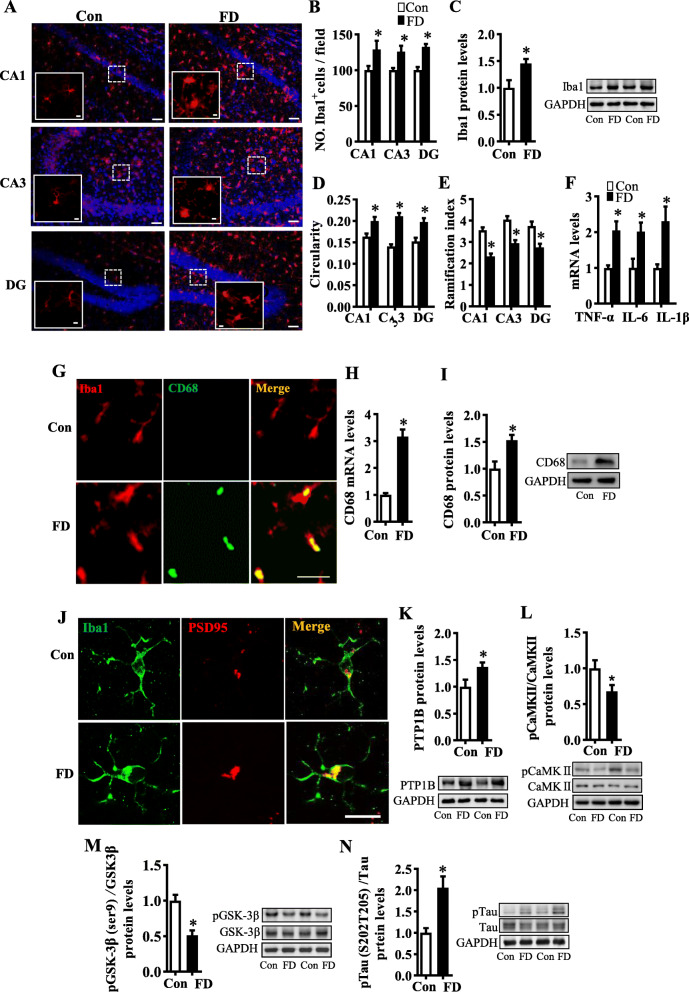
Dietary fiber deficiency for 15 weeks induced microglial synaptic engulfment and neuroinflammation-synapse loss in the hippocampus. **A** The immunofluorescent staining of Iba1 (scale bar: 50 μm), the image capture from the box marked with a dotted line (scale bar: 10 μm). **B** Quantification of Iba1^+^cells numbers in CA1, CA3, and DG of the hippocampus (2 images per mouse, *n* = 6). **C** The protein level of Iba1 in the hippocampus (*n* = 6). **D**, **E** The circularity and ramification index of Iba1^+^ cells (2 images per mouse, *n* = 3). **F** mRNA expression of pro-inflammatory cytokines, TNFα, IL-6, and IL-1β in the hippocampus (*n* = 5). **G** The representative immunofluorescent staining of CD68 in the hippocampus, Scale bar: 25 μm. **H** mRNA expression of CD68 in the hippocampus (*n* = 6). **I** The protein level of CD68 in the hippocampus (*n* = 6). **J** The orthogonal view of the high-resolution confocal image shows the colocalization of Iba1 (green) and PSD95 (red) (Scale bar: 5μm). (K) The protein level of PTP1B (*n* = 6). **L**–**N** The protein level of pCaMKII/CaMKII, pGSK3β/GSK3β, and pTau/Tau in the hippocampus (*n* = 6). Values are mean ± SEM. *p < 0.05 fiber deficiency (FD) group vs. control (Con) group

To address how microglia cells modulate synaptic connectivity, we characterized the morphology of microglia and investigated microglia-synaptic engulfment in FD mice. By immunofluorescent staining with the Iba1 antibody (a microglia-specific calcium-binding protein), we found increased microglia cell number, enlarged microglial cell bodies, and fewer microglial branches in the hippocampal CA1, CA3, and DG regions (Fig. 3A, B), as well as increased Iba1 protein levels (*p* < 0.05, Fig. 3C) in FD mice. Sholl analysis of Iba1^+^ cells revealed increased activated microglia in the FD group, evidenced by an increased circularity index and decreased ramification index (both *p* < 0.05, Fig. 3D, E). Furthermore, we found that the levels of pro-inflammatory cytokines, tumor necrosis factor (TNF)-α, interleukin (IL)-6 and IL-1β were increased in the hippocampus of FD mice (all *p* < 0.05, Fig. 3F), indicating pro-inflammatory M1 activation. The immunofluorescence intensity of CD68 (phagocytic marker) was upregulated in the microglia of the FD group (Fig. 3G) with increased mRNA and protein levels in the hippocampus (both *p* < 0.05, Fig. 3H, I). There were increased PSD-positive puncta enveloped by microglia in the FD group (Fig. 3J), suggesting that dietary fiber deficiency activated microglia and induced the engulfment of synapses in the hippocampus. Following this observation of fiber deficiency-induced neuroinflammation, we found that FD mice had increased protein tyrosine phosphatase 1B (PTP1B) (*p* < 0.05, Fig. 3K), an important mediator which crosslinks inflammation to synaptic alterations [19, 20]. PTP1B can inhibit CaMKII activation [21] and impair the pCaMKII-pGSK3β synaptogenesis pathway resulting in Tau phosphorylation, a critical mark in several neurodegenerative diseases [22–24]. Consistent with these findings, we observed that dietary fiber deficiency decreased levels of pCaMKII and pGSK3β (both *p* < 0.05, Fig. 3L, M) and upregulated pTau (*p* < 0.05, Fig. 3N). Collectively, these findings indicate that fiber deficiency induces microglia activation-mediated synaptic engulfment with alterations of synaptic signaling molecules in the hippocampus.

### Dietary fiber deficiency impairs the intestinal barrier and alters gut microbiota

**Fig. 4:**
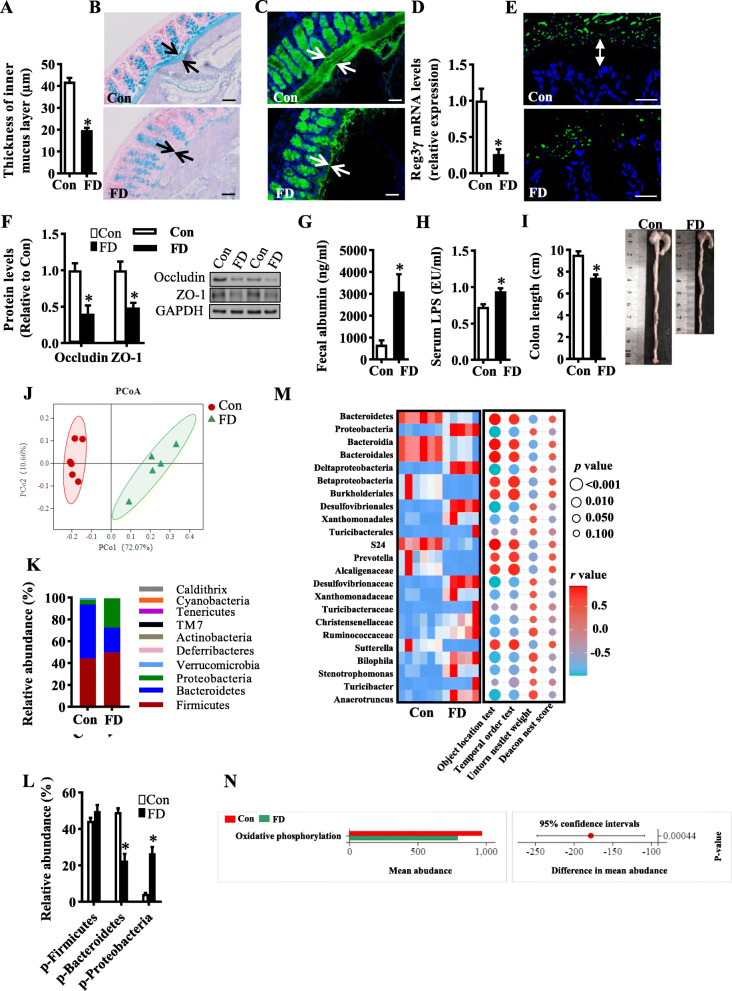
Dietary fiber deficiency for 15 weeks impaired the intestinal barrier and induced intestinal inflammation and microbiota alteration. **A** The quantification of the colonic mucus layer was statistically analyzed (2 sections per animal, *n* = 5). **B** Alcian blue-stained colonic sections were showing the mucus layer (arrows). Opposing black arrows with shafts delineate the mucus layer that was measured. Scale bar: 50 μm. **C** Immunofluorescence images of colonic sections stained with MUC2 (green) and DAPI (blue). Opposing white arrows with shafts delineate the mucus layer. Scale bar: 50 μm. **D** Quantitation of colonic Reg3γ by RT-PCR (*n* = 6). (E) FISH analysis of the colon sections using the general bacterial probe EUB338-Alexa Fluor 488 (green) and nuclear staining DAPI (blue). Arrows indicate the distance between bacteria and epithelium. Scale bar: 10 μm. **F** Protein expression levels of occludin and ZO-1 in the colon (*n* = 5). **G** Fecal albumin concentrations (*n* = 8). **H** Serum endotoxin level (*n* = 10). **I** The quantification of colon length was statistically analyzed (*n* = 9) and representative images of colons. **J** Principal coordinates analysis plot of bray distances of the cecal microbiome. **K**, **L** Composition of abundant bacterial phyla. **M** The association between gut microbiota and cognitive behaviors. Totally, we identified 25 microbiota responsible for discriminating the FD and control groups using linear discriminant analysis (LDA > 2.0). The intensity of color in the middle heatmap (blue to red) indicates the normalized abundance score for each microbiota. The size and color of each point in the scatter plot show the *p* values of Spearman’s correlation (ranging from 6.62e−8 to 0.80) and values of correlation coefficient (ranging from − 0.94 to 0.98) between differential microbiota and cognitive behaviors, respectively. **N** Predicted KEGG functional pathway differences at level 3 inferred from 16S rRNA gene sequences using PICRUSt. Values are mean ± SEM. *p < 0.05 fiber deficiency (FD) group vs. control (Con) group. Abbreviations: p, phylum

Gut homeostasis is important for brain function [11, 12]. We examined whether fiber deficiency perturbed intestinal homeostasis, including intestine barrier integrity, permeability, inflammation, and gut microbiota. Using Alcian blue-staining, we found that the thickness of the colonic mucus was decreased in the FD group compared with the control group (*p* < 0.05, Fig. 4A, B), which was confirmed by the staining of MUC2, the inner layer of mucus (Fig. 4C). Furthermore, the mRNA expression of antimicrobial peptides, Reg3γ, was decreased in the colon tissue of the FD group (*p* < 0.05, Fig. 4D). The distance from microbiota to the epithelial cells was shorter in the FD group (Fig. 4E), indicating bacterial aggregation and mucus invasion. Also, the FD mice showed lower levels of epithelial tight junction proteins, occludin, and zonula occludens-1 (ZO-1) (both *p* < 0.05, Fig. 4F) and higher levels of fecal albumin and serum LPS (both *p* < 0.05, Fig. 4G, H), indicating impaired epithelial barrier integrity and increased gut permeability. Moreover, intestinal inflammation and systemic inflammation were increased in the FD group, as there was an increase in TNFα, IL-6, and IL-1β mRNA expression in the colon (all *p* < 0.05, Figure S1G) and in the serum of FD mice (all *p* < 0.05, Figure S1H). The length of the colon was reduced after the FD diet (Fig. 4I).

Next, gut microbiota diversity and composition in response to fiber deficiency were analyzed using 16S rRNA gene sequencing. The principal component analysis (PCoA) showed relatively separated distribution profiles, suggesting an apparent difference in β-diversity for the microbial community between the FD and the control group (Fig. 4J). In α-diversity, fiber deficiency significantly decreased species richness estimates in Chao1, Ace, and Sob indexes (all *p* < 0.05, Figure S2A-C) but not species diversity in Shannon index (Figure S2D). At the phylum level, the gut microbiota of both control and FD mice was mainly composed of Firmicutes, Bacteroidetes, and Proteobacteria (Fig. 4K). Bacteroidetes were significantly reduced in the FD mice compared with the control group, while Proteobacteria were increased (both *p* < 0.05, Fig. 4L). Using linear discriminant analysis effect size (LEfSe) analysis, we identified that the lower taxa bacteria of phylum Bacteroidetes were also decreased, including class Bacteroidia, order Bacteroidales, family S24-7 and genus *Prevotella* (Fig. 4M and Figure S2E). However, FD mice had increased enrichment of the lower taxa of Proteobacteria, including class Deltaproteobacteria, order Desulfovibrionales and Xanthomonadales, family Desulfovibrionaceae and Xanthomonadaceae, and genera *Bilophila* and *Stenotrophomonas* (Fig. 4M and Figure S2E). In addition, we found that these altered microbiota within Bacteroidetes and Proteobacteria phylum were significantly linked with cognitive indexes in object location, temporal order and nesting behavioral tests (Fig. 4M). These results strongly suggest host-specific alterations of gut microbiota in Bacteroidetes phylum and Proteobacteria phylum are the hallmark of the fiber deficiency mice with cognition decline. Furthermore, using KEGG annotation and functional enrichment, we identified 18 functional categories that exhibited different enrichment levels between the FD and control groups (Figure S2F). The function associated with oxidative phosphorylation was reduced in the FD group (Fig. 4N). The KEGG pathway analysis also indicated that the perturbed gut microbiota by fiber deficiency diet was associated with decreased microbial metabolic capacity (Figure S2F), including folate biosynthesis, one carbon pool by folate, the biosynthesis of glycosphingolipids and the peroxisome, which are involved in the development and function of the nervous system [25–29].

### Gut microbiota alterations and mucus thinning from fiber deficiency occur prior to cognitive impairment.

**Fig. 5:**
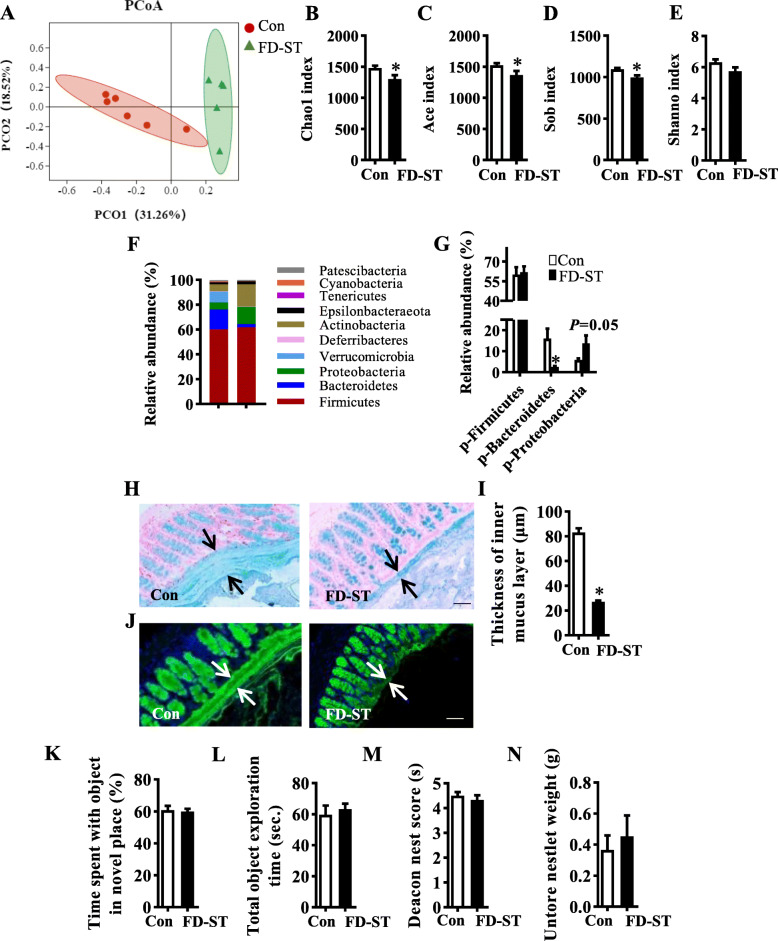
Gut microbiota alteration and thinning mucus, but not cognition decline, were observed after a fiber deficiency diet for 7 days. **A** Principal coordinates analysis plot of bray distances. **B**–**E** The α-diversity of the cecal microbiome is depicted according to Chao1 index (**B**), Ace index (**C**), Sob index (**D**), and Shannon index (**E**). **F**, **G** Composition of abundant bacterial phyla. **H** Alcian blue-stained colonic sections were showing the mucus layer (arrows). Opposing black arrows with shafts delineate the mucus layer that was measured. Scale bar: 50 μm. **I** The quantification of the colonic mucus layer was statistically analyzed (2 sections per animal, *n* = 5). **J** Immunofluorescence images of colonic sections stained with MUC2 (green) and DAPI (blue). Opposing white arrows with shafts delineate the mucus layer. Scale bar: 50 μm. The object location (**K**, **L**) and nest building tests (**M**, **N**) were performed to evaluate the cognition of the mice (*n* = 15). **K** Percentage of time spent with the object in the novel place to total object exploration time. **L** The total object exploration time. **M** The nest score. **N** Untore nestler weight (amount of untore nesting material). Values are mean ± SEM. *p < 0.05 fiber deficiency for short-term (FD-ST) vs. control (Con) group. Abbreviations: p, phylum

To investigate whether gut alteration induced by fiber deficiency preceded the cognition changes, we analyzed gut microbiota and other colon parameters, and the cognitive behaviors of mice with short-term fiber deficiency (FD-ST) for 7 days. We found significant differences in gut microbial profile (Fig. 5A) and species richness (Chao1, Ace, and Sob index) (all *p* < 0.05, Fig. 5B–D) between FD-ST and the control group, but not in species diversity (Shannon index) (Fig. 5E). Furthermore, the short-term FD diet robustly decreased Bacteroidetes phylum and increased the Proteobacteria in statistical trend (Fig. 5F, G) with 11 functional orthologs were altered (Figure S3A). Interestingly, we found short-term fiber deficiency impaired the intestinal mucosa barrier as evidenced by the decreased thickness of colonic mucus stained by Alcian blue and MUC2 antibody (Fig. 5H–J), but did not change colon tight junctions and inflammatory markers (Figure S3B-H). Finally, a short-term FD diet did not alter cognitive behaviors (Fig. 5K–N) and body weight (Figure S3J), but significantly increased energy intake (Figure S3I). Therefore, short-term fiber deficiency alters gut homeostasis, including the microbiota and colon mucus barriers, prior to alterations in cognitive function.

### The reduction of SCFAs induced by fiber deficiency contributes to cognitive impairment

**Fig. 6:**
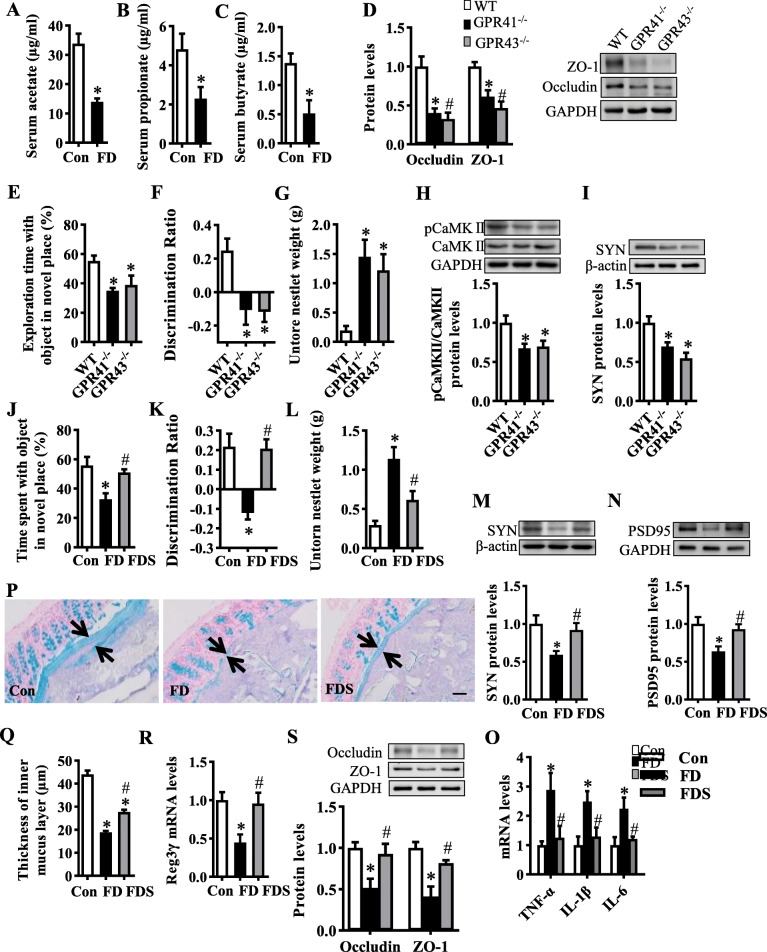
The reduction of short-chain fatty acids by fiber deficiency is critical for cognitive impairment. **A**–**C** The serum levels of acetate, propionate, and butyrate were decreased in mice with the fiber deficiency diet (FD) for 15 weeks (*n* = 6). **D**–**I** The parameters of the gut-brain axis were examined in GPR41^−/−^ and GPR43^−/−^ mice. **D** Protein expression levels of occludin and ZO-1 in the colon (*n* = 5). The object location (**E**), temporal order memory (**F**), and nest-building (**G**) tests were performed (*n* = 8–10). **E** Percentage of time spent with the object in the novel place to total object exploration time. **F** The discrimination ratio. **G** Untore nestler weight (amount of untore nesting material). **H**, **I** Protein levels of pCaMKII/CaMKII and SYN in the hippocampus (*n* = 6). **J**–**S** The parameters of the gut-brain axis in FD mice with SCFAs supplementation (FDS). The object location (**J**), temporal order memory (**K**), and nest building (**L**) tests were performed (*n* = 15). **J** Percentage of time spent with the object in the novel place to total object exploration time. **K** The discrimination ratio. **L** Untore nestler weight. **M**, **N** Protein expression levels of SYN and PSD95 in the hippocampus (*n* = 6). **O** The mRNA expression of pro-inflammatory cytokines, TNFα, IL-1β, and IL-6 in the hippocampus (*n* = 5). **P** Alcian blue-stained colonic sections were showing the mucus layer (arrows). Opposing black arrows with shafts delineate the mucus layer that was measured. Scale bar: 50 μm. **Q** The quantification of the colonic mucus layer was statistically analyzed (2 sections per animal, *n* = 5). **R** Quantitation of colonic Reg3γ by RT-PCR (*n* = 6). **S** Protein expression levels of occludin and ZO-1 in the colon (*n* = 5). Values are mean ± SEM. *p < 0.05 vs. wild-type (WT) or control (Con) groups. ^#^*p* < 0.05 vs. fiber deficiency (FD) group

SCFAs are important metabolites of dietary fiber fermentation by gut microbiota [30]. Here, we found that serum concentrations of acetate, propionate, and butyrate were all decreased by 60–70% in FD mice compared with the control mice (all *p* < 0.05, Fig. 6A–C). To determine the role of SCFAs in FD diet-increased intestinal permeability, we employed SCFAs receptor knockout mice, GPR41^−/−^ and GPR43^−/−^ mice. These mice exhibited decreased levels of tight junction proteins, ZO-1 and occludin, in the colon tissue (all *p* < 0.05, Fig. 6D). Furthermore, the cognitive index was decreased in object location, temporal order memory, and nesting behavioral tests of GPR41^−**/**−^ and GPR43^−**/**−^ mice (Fig. 6E–G, Figure S4A-C) with lower levels of synaptic proteins pCaMKII and SYN in the hippocampus (Fig. 6H, I). Therefore, fiber deficiency-induced SCFAs deficit and inactivation of SCFA receptors may serve as mediating factors between intestinal dysfunction and cognitive impairment.

To determine whether the decreased levels of SCFAs could be attributed to alterations of the gut-brain axis following fiber deficiency, we treated FD mice with SCFAs (FDS) for 15 weeks. Supplementation with SCFAs significantly increased object location memory, temporal order memory, and the ability to build a nest when compared to the FD group without SCFA supplementation (all *p* < 0.05, Fig. 6J–L and Figure S5A-C). SCFAs restored the FD-diet induced loss of synaptic proteins SYN and PSD95 in the hippocampus (Fig. 6M, N). Furthermore, supplementation with SCFAs inhibited the microglia activation (Figure S5D-H), phagocytosis of microglia to the synapse (Figure S5I-K), and neuroinflammation (Figure 6O), otherwise evident in FD mice. Also, SCFAs partially restored the reduction in the thickness of colonic mucus (Fig. 6P, Q; Figure S6A and B) and improved colonic antimicrobial properties (Fig. 6R), epithelial tight junctions (Fig. 6S) and permeability (Figure S6C and D), and colonic and systemic inflammation (Figure S6E-G) compared with FD mice. These findings indicate that supplementation with SCFAs prevents the gut-brain axis dysfunction induced by a fiber-deficient diet.

## Discussion

People in industrialized nations currently consume approximately half the recommended amount of dietary fiber [4–6]. However, the ways in which the brain is affected by fiber deficiency remains wholly understudied. In the present study, we provided the first evidence that dietary fiber deficiency leads to cognitive dysfunction. Furthermore, we demonstrate in mice that this is associated with alterations of hippocampal synaptic ultrastructure and proteomic profiles (including dysregulation of the CaMKIId interacted synaptic proteins) and neuroinflammation-mediated synaptic loss. The FD mice also exhibited gut dyshomeostasis, including mucosa barrier impairment and microbiota dysbiosis (disturbances of the phylum Bacteroidetes and Proteobacteria). In addition, the deficit of the intestinal mucus barrier and microbial deviation from the normal state occurred prior to the cognitive decline, suggesting the earlier response of gut dyshomeostasis to fiber deficiency. Notably, the deficit of SCFAs (metabolites of fiber by gut microbiota) was involved in mediating the gut-brain dysfunction in FD mice; SCFA supplementation prevented the leaky gut and cognitive decline of FD mice and the SCFA receptor knockout (GPR41^−/−^, 43^−/−^) mice showed abnormal gut-hippocampal synaptic axis.

A clinical study of 65 children aged between 7 and 9 years showed that dietary fiber intake was positively correlated with congruent or incongruent accuracy [31]; however, the mechanisms linking dietary fiber with cognitive function remain elusive. In this study, we revealed that the fiber-deficient diet altered the synaptic ultrastructure and proteome in the mouse hippocampus, which is implicated in the neuropathology of cognition decline, including in object location memory, temporal order memory, and the ability to perform daily living activities. Specifically, the proteome of cholinergic synapses and dopamine synapses was affected by the FD diet. Cholinergic and dopaminergic neurotransmission is important for hippocampal-dependent synaptic plasticity and memory regulation [32, 33]. For example, cholinergic hypofunction occurs at the prodromal stages of AD-associated cognitive decline [34, 35]. Furthermore, the injection of a dopamine D1-receptor antagonist can block the novel object preference behavior in rodents [32]. In the present study, CaMKIId was decreased in the FD animals, which is a core protein among cholinergic and dopaminergic synapse pathways. It is likely that the alteration of CaMKIId and its associated synaptic proteins in the PPI network contribute to FD-induced cognitive decline, as previous research has shown that persistent expression of CaMKIId in the hippocampus is required for long-lasting memory storage of novel object recognition tasks in rodents [36]. Furthermore, GAP43 and SV2C, synaptic markers of cognitive decline in the brains of dementia patients [18], were revealed here to interact with CaMKIId and were decreased in the hippocampus of FD mice. Therefore, altering these synaptic proteins may be a critical predictive molecular fingerprint for dietary fiber deficiency-associated cognitive decline.

PTP1B is an important mediator crosslinking neuroinflammation and synapse impairment [19, 20]. For example, the pro-inflammatory cytokine TNFα or endotoxins increase PTP1B in the mouse brain [19, 37]. Subsequently, PTP1B can inhibit CaMKII activation [21]. pCaMKII can increase pGSK3β to inhibit Tau phosphorylation, a key process of neurodegenerative disease [22–24]. We found that not only the main isoform of CaMKII (CaMKIId) was down-regulated, but also that the CaMKII downstream cascade pCaMKII-pGSK-pTau was dysregulated in FD mice. Therefore, these findings suggest that hippocampal microglia activation and neuroinflammation caused by fiber deficiency may induce synaptic ultrastructure impairment and aberrant synaptic signaling cascades. Emerging research suggests that the dysregulation of gut homeostasis induces neuropathology by affecting neuroimmunological and synaptic homeostasis. In the colitis mouse model induced by dextran sodium sulfate, elevated frequencies of inflammatory M1-like microglia, and increased release of pro-inflammatory cytokines are observed in the hippocampus [38]. In clinical studies, patients with inflammatory bowel disease present with impaired cognitive function and decreased hippocampal activity, as assessed by magnetic resonance imaging [39, 40]. In line with this evidence, we found that fiber deficiency impaired colonic homeostasis, including the loss of barrier integrity and inflammation, which is concomitant with the appearance of hippocampal neuroinflammation and synaptic impairment, indicating a perturbed gut-brain axis.

Serum LPS level was significantly increased in the FD mice. This might be attributed to gut microbiota alteration, intestinal barrier dysfunction, and increased permeability. It is reported that the LPS levels are increased threefold in the blood and two- or threefold in the brain of AD patients [13, 41]. Previously, systemic exposure of LPS (a single intraperitoneal injection) induces microglial activation and increases pro-inflammatory cytokines in the hippocampus of mice [42]. Our previous study also showed that intracerebroventricular injection of LPS in mice induced impairments in spatial learning and memory, loss of synapse-associated proteins (SYN and PSD95) and microglia activation in the hippocampus [43]. In the present study, we found that the FD mice had activated microglia with over-expression of phagocytosis marker CD68, which promoted the synapses engulfment in the hippocampus. As LPS is found to cross the blood-brain barrier (BBB) [44], we speculate that LPS activates microglia that engulfs synaptic proteins and thus alters synaptic proteome in the FD mice. Furthermore, our previous in vitro study shows that supernatant from LPS-stimulated microglia increases NF-κB p65 and decreases cell viability of hippocampal neurons [43]. NF-κB promotes the expression of PTP1B [45], which can inhibit the pCaMKII-pGSK3β synaptogenesis pathway [21]. Therefore, it is plausible that the FD diet increases LPS production, which penetrates the thinned gut barrier and crosses BBB. In the FD mouse brain, LPS promotes the inflammatory-neurodegeneration cascade PTP1B-pCaMKII-pGSK3β. Together with phagocytosis of activated microglia, enhanced PTP1B signaling in the FD mice induces alterations in synaptic substrates in the hippocampus, and ultimately leads to cognitive decline.

As a component of the gut-brain axis, the gut microbiota plays a vital role in cognitive health [8]. Previously, a fiber-deficient diet for 7 weeks in mice results in a decreased abundance of 60% taxa in the gut microbiota [46]. Here, we report that most of the taxa decreased by fiber deficiency were from phylum Bacteroidetes (the dominant gut microbial phyla) and its lower levels, such as Bacteroidia at class, Bacteroidales at order, S24-7 at family, and *Prevotella* at genus. It is reported that Bacteroidetes genomes encode various enzymes involved in the metabolism and acquisition of polysaccharides from dietary fiber [47]. Therefore, our results revealed that fiber-deficient diet disrupts the growth of Bacteroidetes and reduces its abundance. Bacteroidetes alone or in conjunction with other gut microbiota benefit their host mucus barrier [48]. For instance, the transplantation of co-colonization of *Bacteroides thetaiotaomicron* with a common gut microbiome into germ-free mice increases the gene expressions of the enzymes required for the synthesis of mucosal glycans in colon tissue [49]. Therefore, decreased levels of Bacteroidetes could reduce the production of mucosal glycans and, in turn, thin the mucus layer overlying the intestinal epithelium, thereby resulting in epithelial barrier damage. Moreover, we also found that fiber deficiency caused a composition shift of gut microbiota by increasing the abundance of phylum Proteobacteria, a major source of antigen LPS [50]. In clinical studies, the proportion of Proteobacteria is higher in individuals with mild cognitive impairment [51]; however, phylum Bacteroidetes and order Bacteroidales are lower in the elderly with impaired cognitive function [52, 53] and AD patients [54]. Our study suggested that fiber-deficient diet increases phylum Proteobacteria that enhances the LPS production. As above mentioned, increased LPS may cross the leaky gut (gut barrier impairment and increased permeability) and BBB, and then activate neuroinflammation that results in cognitive impairment. In particular, we found that short-term (7 days) fiber-deficient diet only induced the alteration of gut microbiota and colon mucus barrier deficit but not cognitive decline, suggesting cognitive impairment is the consequence of the alterations of gut microbiota composition.

Interestingly, our findings revealed that reduced fiber content in the diet resulted in lower levels of three types of SCFAs; acetate, propionate and butyrate, which are fermented metabolites of dietary fiber by the microbiota. SCFAs act via GPCRs, i.e., GPR41 and GPR43 receptors, which are highly expressed in gut epithelial and innate immune cells [55, 56]. Previously, GPR41^−/−^ and GPR43^−/−^ mice showed increased susceptibility to DSS-induced colitis or the infection by *Citrobacter rodentium* [57–59]. Here, we showed that GPR41^-/-^ and GPR43^-/-^ mice exhibited a leaky gut and cognitive decline, indicating that SCFA signalling through GPR41 and 43 plays an important role in the gut-brain axis. In line with these findings, our results also showed that SCFA supplementation restored fiber-deficient diet-reduced mucus thickness and MUC2 level and decreased antimicrobial peptides Reg3γ in the colon of mice. Our result is supported by a previous in vitro study, which showed that SCFAs increase the MUC2 protein level in intestinal epithelial cells [60]. Furthermore, we found that SCFA supplementation rescued the leaky gut (an increase of tight junction proteins and decrease in gut permeability), neuroinflammation, synaptic protein loss, and abnormal cognitive behaviors of the FD mice. Taken together, these findings indicate that decreased SCFAs due to fiber deficiency may lead to an inactivation of GPR41 and GPR43 and a dysregulated gut-brain axis contributing to cognitive decline. In addition, in the present study, the KEGG pathway analysis also indicates that the perturbed gut microbiota by fiber-deficient diet was associated with decreased oxidative phosphorylation, the most abundant metabolic pathway in the host. It is known that colonocyte metabolism is directed toward oxidative phosphorylation, resulting in a hypoxic environment in the intestinal tract [61], which is beneficial to the growth of obligate anaerobes to ferment the dietary fiber [61]. In FD mice, reduced oxidative phosphorylation may lead to a hyper-oxidative environment in the gut and further reduction of SCFAs.

Cognitive decline is the major clinical manifestation in patients with AD and dementia. AD models had been widely used to investigate the neuropathology underlying cognitive impairment. However, AD and dementia are multifactorial and involved in several etiopathogenic mechanisms and modifiable risk factors [62, 63]. In recent years, research demonstrates that the disturbances along the microbiota-gut-brain axis significantly contribute to the pathogenesis of AD [62]. For example, the microbiota composition and diversity are altered and SCFAs level is reduced in the AD mouse model with ultrastructural abnormalities of the intestine [64]. Probiotic formulation of lactic acid bacteria and bifidobacterial counteracts cognitive decline and brain damage in the AD mouse model with a reduction of plasma pro-inflammatory cytokines [65]. In clinical studies, it is reported that there are significant differences in richness, diversity, and composition of the gut microbiome in AD patients and individuals with mild cognitive impairment compared to mentally healthy individuals of the same age [51–54]. In addition, a population-based (17,420 men and women) cohort study demonstrates a significant association between inflammatory bowel disease and subsequent development of dementia [66]. Furthermore, the Lancet commission reported that more than one-third of dementia cases could be prevented through addressing lifestyle factors, including diet [67]. Excess energy intake and consequent metabolic disorders, such as obesity and diabetes, have adverse effects on cognition and are the established risk factors for the development and progression of AD [63, 68]. In the present study, the deteriorating effect of dietary fiber deficiency on cognitive function in the mouse model is more closely to the natural living state of human beings experienced with multifactorial lifestyle risk factors and enteropathogenic statuses, such as Western-pattern diet, overeating, adiposity as well as gut microbiota disturbance, alteration of metabolites of gut microbiota (LPS and SCFAs) and intestinal inflammation.

Energy intake was increased in the FD diet-fed group both in the short-term and long-term experiment, suggesting that the satiety was decreased. High fiber intake, such as β-glucan, lupin kernel fiber, and rye, possess satiety-enhancing effects [69]. Our previous clinical and rodent studies have shown that dietary fiber, oat β-glucans increases colon L cell-derived satiety hormone peptide YY (PYY) to suppress appetite [70, 71]. It is reported that SCFAs, the metabolites of fiber, stimulate PYY production in human enteroendocrine cells [72]. Furthermore, dietary fiber increases satiety by activation of the gut-hypothalamic (PYY-NPY) axis in mice [71]. These findings suggest that dietary fiber regulates the gut-brain axis to increase satiety, while the deficiency of fiber intake may reduce satiety and increase appetite and energy intake. It is reported that excessive energy intake has adverse effects on cognition in humans and rodent studies, while energy restriction decreases neuroinflammation and increases synaptic plasticity associated proteins [68]. Indeed, we found that the amount of energy intake was positively correlated with body weight and adipose tissue, and negatively with cognitive parameters (Table S2 in Supporting Information). Therefore, the excess energy intake and adverse metabolic parameters induced by the FD diet may contribute to cognition impairment. Importantly, we found that the FD diet induced alterations of gut microbiota, gut barrier disruption, hippocampal inflammation, and synaptic ultrastructure abnormalities. Overall, apart from regulating satiety via the gut-brain (PYY-hypothalamic NPY) axis, fiber deficiency also induces the disturbance in the gut-hippocampus axis for the development of cognition decline.

Using 32% and 64% sucrose in fiber-deprived diets in Sonnenburg et al.’s study and Desai et al.’s study, the mice exhibit gut microbiota alteration and degradation of the colonic mucus barrier [46, 73]. For example, in Sonnenburg et al.’s study, the fiber deficiency diet with 32% sucrose for 7 weeks, decreases the abundance of taxa in the gut microbiota of mice with the most of the lost taxa from the Bacteroidales order [46]. In Desai et al.’s study, after the fiber-free diet with 64% sucrose for 6 weeks, the colonic mucus of the mice is five to six times thinner than the control mice fed the Con diet [73]. Consistently, in the present study, the decreased richness of microbiota (Chao1, Ace, and Sob index), decreased Bacteroidetes phylum and decreased mucus thickness were observed not only after fiber deficiency diet for 15 weeks, but also in short-term study after 7 days. In the above studies including ours, to mimic the Western-style diet (high simple carbohydrates and low fiber), the fiber-deprived diets utilize sucrose for replacing the fiber and microbiota-accessible carbohydrate in the control diet. Therefore, the higher content of sucrose and low fiber diets induced an early stage gut microbiota shift and mucosa thinner.

Research showed that consumption of 11% sucrose solution for 4 weeks did not increase neuroinflammation in rats [74], while a prolonged high sucrose intake (5% sucrose solution) for 12 weeks provoked systemic and neuroinflammation in rats [75]. In addition, Hsu’s study showed that high sucrose intake for 4 weeks moderately impaired hippocampal-dependent spatial learning and memory in adolescent rats [74]. Similarly, our short-term study with FD diet for 7 days did not show changes in cognitive function, suggesting that high content of sucrose in the FD diet did not acutely decrease the cognition. Therefore, these findings indicate that sucrose-induced neuroinflammation and cognition alteration were chronic effects. Again, in the present study, after the chronic FD diet for 15 weeks, the mice had cognition decline, neuroinflammation and microglial engulfment of synapses in the hippocampus. Furthermore, gut microbiota dysbiosis (decreased Bacteroidetes and increased Proteobacteria) were significantly associated with the impaired cognitive behaviors of FD mice. Overall, these findings indicate that sucrose as simple carbohydrates in the fiber deficiency diet contributed to gut dyshomeostasis initially and thereafter systemic neuroinflammation and cognition impairment.

## Conclusions

**Fig. 7:**
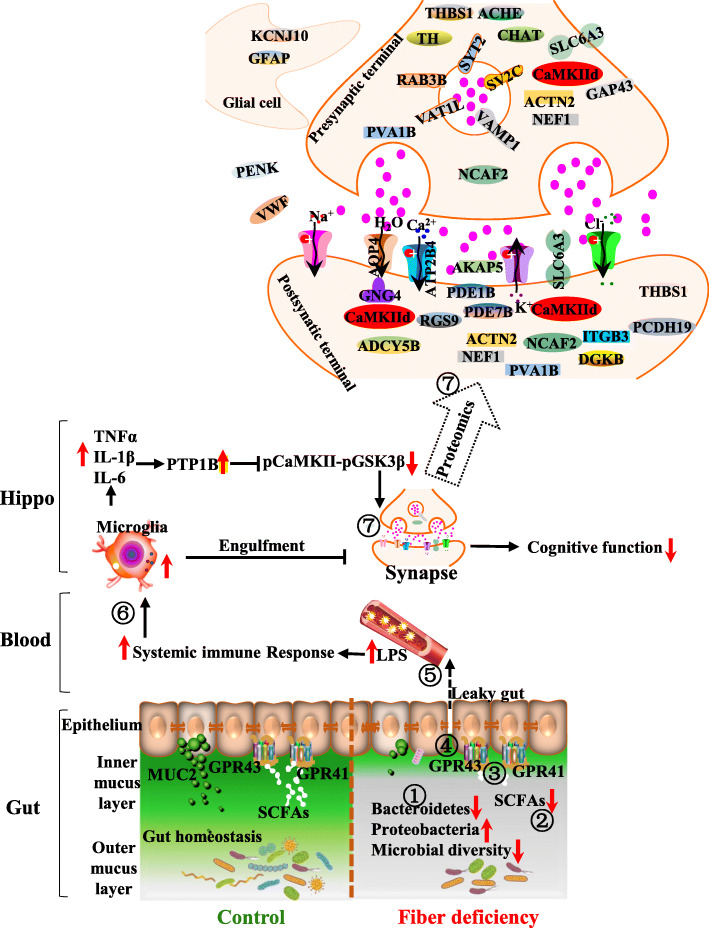
The dietary fiber deficiency disturbs the gut-hippocampal axis attributable to cognition decline. Alteration of gut microbiota and the deficit of short-chain fatty acids (SCFAs) due to fiber deficiency promote adverse consequences on cognition (Step 1–7). Dietary fiber deficiency results in microbiota alteration (the decrease of microbial diversity and Bacteroidetes, and increase of Proteobacteria) (**1**) and the deficit of SCFAs and their associated inactivation of GPR41 and GRP43 (**2** and **3**), thereby attributable to mucus and epithelial barrier deficit (**4**), over translocation of LPS (the out membrane of Proteobacteria) into the blood circulation (**5**), systemic inflammatory tone to activate microglia (**6**) and thereafter synapse engulfment and synaptic proteins alteration in the hippocampus (Hippo) (**7**). Therefore, dietary fiber deficiency leads to an adverse impact on brain function via the gut-hippocampal axis

The present study identified a robust phenotype of cognitive decline in a mouse model of chronic dietary fiber deficiency featuring an altered gut-hippocampal axis. Notably, the gut microbiota dysbiosis and the reduction of SCFAs suggest an interplay between dietary fiber deficiency and host gut-brain axis alteration (Fig. 7). Our findings indicate an adverse impact of dietary fiber deficiency on brain function and highlight a nutritional strategy of increased fiber intake to reduce the risk of diet-associated cognitive decline and neurodegenerative diseases.

## Methods

### Animals

Wild-type (WT) C57BL/6J male mice (10 weeks old) were obtained from the Experimental Animal Center of Xuzhou Medical University (SCXK_Su_2015-0009). The GPR41^−/−^, and GPR43^−/−^ mice and their littermates were obtained by intercrossing GPR41^+/−^ and GPR43^+/−^ mice (GPR41^+/−^, C57BL/6J–Gpr41/BRL, Stock No. KO-0005; GPR43^+/−^, C57BL/6J–Gpr43/BRL, Stock No. KO-0006; Shanghai Bioray Laboratories Inc., China). The mice were housed in environmentally controlled conditions (temperature 22 °C, 12-h light/dark cycle). After acclimatization to the laboratory conditions for 1 week, the mice were used for experiments approved by the Institutional Animal Care Committee of Xuzhou Medical University following the Chinese Council on Animal Care Guidelines.

### Study design and experimental timeline

#### Dietary fiber deficiency experiment

C57BL/6J male mice (11 weeks old) were randomly divided into two groups (*n* = 30 per group) fed either the control (Con) diet (LabDiet 5010) or fiber deficiency (FD) diet. Each group of mice was sacrificed at two-time points: 15 weeks (long-term) or 7 days (short-term) (*n* = 15 per subgroup). The Con diet is a plant polysaccharide-rich diet containing the microbiota-accessible carbohydrate derived from a diverse source of plants, including corn, soybean, wheat, oats, alfalfa, and beet. The reported neutral and acid detergent fiber content of the Con diet is 20% by weight. The FD diet is a low microbiota-accessible carbohydrate diet modified based on Harlan TD.86489, as described previously [46]. The FD diet contains cellulose (5% by weight, low accessibility by gut microbiota) and non-microbiota accessible carbohydrates such as sucrose (31% by weight) and corn starch (31% by weight) (detailed in table S1).

#### Gpr41 and Gpr43 knockout mice experiment

GPR41^−/−^, GPR43^−/−^ mice and WT littermates (*n* = 8–10) were fed the Con diet for 26 weeks, so the mice were sacrificed at the same age as the mice in the long-term dietary fiber deficiency experiment.

#### SCFAs supplementation experiment

The C57BL/6J mice (11 weeks old) were randomly divided into three groups (*n* = 15 per group): (1) the Con diet group, (2) the FD diet group, (3) the SCFAs (FDS) group consuming FD diet and drinking the water with the cocktail of SCFAs mixture (67.5 mM sodium acetate, 40 mM sodium butyrate, and 25.9 mM sodium propionate) [11, 76] for 15 weeks.

After the intervention in the above three experiments, cognitive behavior tests were performed. After 3 days following the last test, the mice were sacrificed using CO_2_ asphyxiation. Blood, cecum content, colon, and brain tissues were immediately collected for further investigations.

### Mass-spectrometry-based proteomics

Mouse hippocampus was ground by liquid nitrogen into cell powder and processed with protein extraction, trypsin digestion, labeling, high-performance liquid chromatography fractionation, liquid chromatography-tandem mass spectrometry (LC-MS/MS) analysis. The resulting LC-MS/MS data were processed using the Mascot search engine (v.2.3.0). Bioinformatics for the result of proteomic was performed including, Gene Ontology (GO) proteome annotation using the UniProt-GOA database (www. http://www.ebi.ac.uk/GOA/); The Kyoto Encyclopedia of Genes and Genomes (KEGG) analysis according to the KEGG website (http://www.genome.jp/kegg/). All protein name identifiers were searched against the STRING database (https://stringdb.org/, v.10.5) for protein-protein interactions (PPI).

### Cecal microbiota analysis (16S rRNA gene sequencing)

The microbiomes were analyzed at the Illumina sequencing platform by Genedenovo Biotechnology Co., Ltd. (Guangzhou, China). High-quality reads for bioinformatics analysis were selected, and all of the valid reads from all samples were clustered into operational taxonomic units (OTUs) based on 97% sequence similarity. The variation between the experimental groups (β-diversity) was assessed with principal coordinate analysis (PCoA) plots. Linear discriminant analysis coupled with effect size (LEfSe) was performed with LEFSE software.

### Immunohistochemistry

Frozen brain (hippocampus) sections in 20 μm were incubated with the primary antibodies, including antibody against Iba1, CD68, and PSD95, followed by the incubation with the secondary antibodies and DAPI. The microglia labeled by Iba1 and CD68 were imaged with a fluorescence microscope (OLYMPUS IX51). The sections double-labeled for Iba1 and PSD95 were visualized by a 63× oil immersion objective in stacks (z-step 0.1 μm) using a Leica SP8 confocal microscope system (Leica, Germany).

### Mucus thickness measurement and bacteria localization in the colon

The colon samples were embedded in paraffin, cut into thin sections (5 μm), and performed by Alcian blue staining as previously published [73]. The mucus thickness was then measured (10 measurements per section/2 sections per animal/5 animals per group) using ImageJ after cross-validation using anti-MUC2 staining. Fluorescence in situ hybridization (FISH) was used to stain bacteria localization at the intestinal mucosa surface as previously described [77].

See “Additional files” section for detailed information of procedures (“behavior”, “mass-spectrometry-based proteomics”, “transmission electron microscopy”, “immunohistochemistry”, “western blotting”, “quantitative RT-PCR”, “cecal microbiota analysis”, “measurement of serum cytokines, LPS and SCFAs”, “mucus thickness measurement and bacteria localization in the colon”).

### Statistical analysis

Data were analyzed using the statistical package SPSS (Version 20, Chicago, USA). After data were tested for normality, the Student’s *t* test was used to compare two groups. For the comparisons of more than two groups, a one-way analysis of variance (ANOVA) was used, followed by the post hoc Tukey-Kramer test for multiple comparisons. A *p* value of < 0.05 was considered to be statistically significant. An α level of 0.05 (two-tailed) was set for significance. The value was considered as an outlier if it had more than two standard deviations from the mean. Sample size, number of replicates, and statistical tests are reported in the “Additional files” section or figure legends.

## Supplementary Information

**Additional file 1: Fig. S1.** Dietary fiber deficiency for 15 weeks impaired cognition and increased colonic and systemic inflammation. Total object exploration time in the object location test (A) and temporal order memory tests (B). (C) Deacon nest score in nest building tests (n=15). (D) Average energy intake (n=15). (E) Body weight at 15 week (n=15). (F) Fat pad weight (n=9). (G) mRNA expression levels of TNFα, IL-1β and IL-6 in the colon (n=5). (H) TNF-α, IL-1β, and IL-6 levels in the serum (n=10). Values are mean ± SEM. *p < 0.05 fiber deficiency (FD) group vs. control (Con) group. **Fig. S2.** Dietary fiber deficiency altered gut microbiota. (A-D) The α-diversity of the cecal microbiome among two groups depicted according to Chao1 index (A), Ace index (B), Sob index (C) and Shannon index (D). (E) Cladogram generated from linear discriminant analysis (LDA) (n=5-6). (F) Predicted KEGG functional pathway differences at level 3 inferred from 16S rRNA gene sequences using PICRUSt. Values are mean ± SEM. *p < 0.05 fiber deficiency (FD) group vs. control (Con) group. **Fig S3.** Dietary fiber deficiency for 7 days altered gut microbiota, but not colon tight junctions and inflammation. (A) Predicted KEGG functional pathway differences at level 3 inferred from 16S rRNA gene sequences using PICRUSt (n=5-6). (B-D) Protein levels of occludin and ZO-1 in the colon (n=6). (E) The quantification of colon length was statistically analyzed (n=9) with representative images of colons. (F-H) mRNA expression levels of TNFα, IL-1β and IL-6 in the colon (n=6). (I) Average energy intake (n=15). (J) Body weight at 1 week (n=15). Values are mean ± SEM. *p < 0.05 fiber deficiency for short-term (FD-ST) group vs. control (Con) group. **Fig. S4.** The cognitive behavior in the GPR41-/- and GPR43-/-mice. Total object exploration time in the object location test (A) and temporal order memory tests (B). (C) Deacon nest score in nest building tests (n=8-10). Values are mean ± SEM. *p < 0.05 vs. wild type (WT) mice. **Fig. S5.** SCFAs supplementation prevented FD-induced cognitive decline and microglia activation. Total object exploration time in the object location test (A) and temporal order memory tests (B). (C) Deacon nest score in nest building tests (n=15). (D) The immunofluorescent staining of Iba1 (Scale bar: 50μm), the image capture from the box marked with a dotted line (Scale bar: 10μm). (E) Quantification of Iba1+cells numbers in CA1, CA3 and DG of the hippocampus (2 images per mouse, n=6). (F) The protein level of Iba1 in the hippocampus (n=6). (G and H) The circularity and ramification index of Iba1+ cells (2 images per mouse, n=3). (I) mRNA expression level of CD68 (n=6). (J) The representative immunofluorescent staining of CD68 in the hippocampus, Scale bar: 25μm. (K) The orthogonal view of the high-resolution confocal image shows the colocalization of Iba1 (green) and PSD95 (red) (Scale bar: 5μm). Values are mean ± SEM. *p < 0.05 vs. control (Con) group. #p < 0.05 vs. fiber deficiency (FD) group. The FD mice with SCFAs supplementation: FDS. **Fig. S6.** SCFAs supplementation prevented FD-induced intestinal integrity impairment, endotoxemia and systemic inflammation. (A) Immunofluorescence images of colonic sections stained with Anti-MUC2 antibody and DAPI. Opposing white arrows with shafts delineates the mucus layer. Scale bar: 50μm (B) FISH analysis of sections of the colon using the general bacterial probe EUB338-Alexa Fluor 488 (green), and nuclear staining DAPI (blue). Arrows indicate the distance between bacteria and epithelium. Scale bar: 20μm. (C) Fecal albumin concentrations (n=8). (D) Serum LPS endotoxin level (n=10). (E) mRNA expression levels of TNFα, IL-1β and IL-6 in the colon (n=5). (F) The quantification of colon length was statistically analyzed (n=9) and representative images of colons. (G) TNF-α, IL-1β and IL-6 levels in the serum (n=10). Values are mean ± SEM. *p < 0.05 vs. control (Con) group. #p < 0.05 vs. fiber deficiency (FD) group. The FD mice with SCFAs supplementation: FDS. **Table S1.** Composition of the control (Con) and fiber deficient (FD) diets. **Table S2.** Pearson correlations between energy intake and metabolic and behavior parameters

## Data Availability

The sequencing data of the 16S rRNA gene in this study are available in the Sequence Read Archive (SRA) under project number PRJNA724800. The mass spectrometry proteomics data are available via ProteomeXchange with identifier PXD025638.

## Abbreviations

AD: Alzheimer’s disease
ANOVA: One-way analysis of variance
FD: Fiber deficiency
FISH: Fluorescence in situ hybridization
GO: Gene Ontology
GPCRs: G protein-coupled receptors
KEGG: The Kyoto Encyclopedia of Genes and Genomes
LC-MS/MS: Liquid chromatography-tandem mass spectrometry
LEfSe: Linear discriminant analysis coupled with effect size
LPS: Lipopolysaccharide
OTUs: Operational taxonomic units
PCoA: Principal coordinate analysis
PPI: Protein-protein interactions
PSD95: Postsynaptic density protein 95
SCFA: Short-chain fatty acid
SYN: Synaptophysin
