# Supplementary material for: A fiber-deprived diet causes cognitive impairment and hippocampal microglia-mediated synaptic loss through the gut microbiota and metabolites
Source: Microbiome. 2021 Nov 11;9:223. doi: 10.1186/s40168-021-01172-0 (PMC8582174; doi:10.1186/s40168-021-01172-0)
Supplement: Supplementary file 2 — Additional file 1: Fig. S1. Dietary fiber deficiency for 15 weeks impaired cognition and increased colonic and systemic inflammation. Total object exploration time in the object location test (A) and temporal order memory tests (B). (C) Deacon nest score in nest building tests (n=15). (D) Average energy intake (n=15). (E) Body weight at 15 week (n=15). (F) Fat pad weight (n=9). (G) mRNA expression levels of TNFα, IL-1β and IL-6 in the colon (n=5). (H) TNF-α, IL-1β, and IL-6 levels in the serum (n=10). Values are mean ± SEM. *p < 0.05 fiber deficiency (FD) group vs. control (Con) group. Fig. S2. Dietary fiber deficiency altered gut microbiota. (A-D) The α-diversity of the cecal microbiome among two groups depicted according to Chao1 index (A), Ace index (B), Sob index (C) and Shannon index (D). (E) Cladogram generated from linear discriminant analysis (LDA) (n=5-6). (F) Predicted KEGG functional pathway differences at level 3 inferred from 16S rRNA gene sequences using PICRUSt. Values are mean ± SEM. *p < 0.05 fiber deficiency (FD) group vs. control (Con) group. Fig S3. Dietary fiber deficiency for 7 days altered gut microbiota, but not colon tight junctions and inflammation. (A) Predicted KEGG functional pathway differences at level 3 inferred from 16S rRNA gene sequences using PICRUSt (n=5-6). (B-D) Protein levels of occludin and ZO-1 in the colon (n=6). (E) The quantification of colon length was statistically analyzed (n=9) with representative images of colons. (F-H) mRNA expression levels of TNFα, IL-1β and IL-6 in the colon (n=6). (I) Average energy intake (n=15). (J) Body weight at 1 week (n=15). Values are mean ± SEM. *p < 0.05 fiber deficiency for short-term (FD-ST) group vs. control (Con) group. Fig. S4. The cognitive behavior in the GPR41-/- and GPR43-/-mice. Total object exploration time in the object location test (A) and temporal order memory tests (B). (C) Deacon nest score in nest building tests (n=8-10). Values are mean ± SEM. *p < 0.05 vs. wild type [file 40168_2021_1172_MOESM2_ESM.docx]

**A fiber-deprived diet causes cognitive impairment and hippocampal microglia-mediated synaptic loss through the gut microbiota and metabolites**

Hongli Shi^1#^, Xing Ge^1#^, Xi Ma^3#^, Mingxuan Zheng^1^, Xiaoying Cui^4^, Wei Pan^1^, Peng Zheng^2^, Xiaoying Yang^1^, Peng Zhang^1^, Minmin Hu^1^, Tao Hu^1^, Renxian Tang^1^, Kuiyang Zheng*****^1^, Xu-Feng Huang*****^2, 1^, Yinghua Yu*****^1^

**Affiliations**

^1^Jiangsu Key Laboratory of Immunity and Metabolism, Department of Pathogen Biology and Immunology, Xuzhou Medical University, Xuzhou, Jiangsu 221004, China;

^2^Illawarra Health and Medical Research Institute (IHMRI) and School of Medicine, University of Wollongong, NSW 2522, Australia;

^3^State Key Laboratory of Animal Nutrition, College of Animal Science and Technology, China Agricultural University, Beijing, 100193, China;

^4^Queensland Brain Institute, The University of Queensland, St Lucia, QLD 4113, Australia.

**Fig. S1**


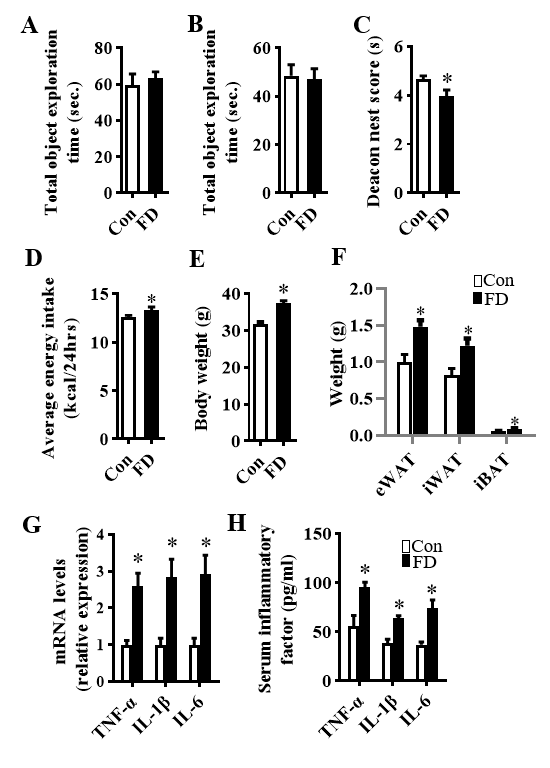


**Fig. S1. Dietary fiber deficiency for 15 weeks impaired cognition and increased colonic and systemic inflammation.** Total object exploration time in the object location test (A) and temporal order memory tests (B). (C) Deacon nest score in nest building tests (n=15). (D) Average energy intake (n=15). (E) Body weight at 15 week (n=15). (F) Fat pad weight (n=9). (G) mRNA expression levels of TNFα，IL-1β and IL-6 in the colon (n=5). (H) TNF-α, IL-1β, and IL-6 levels in the serum (n=10). Values are mean ± SEM. **p* < 0.05 fiber deficiency (FD) group vs. control (Con) group.

**Fig. S2**


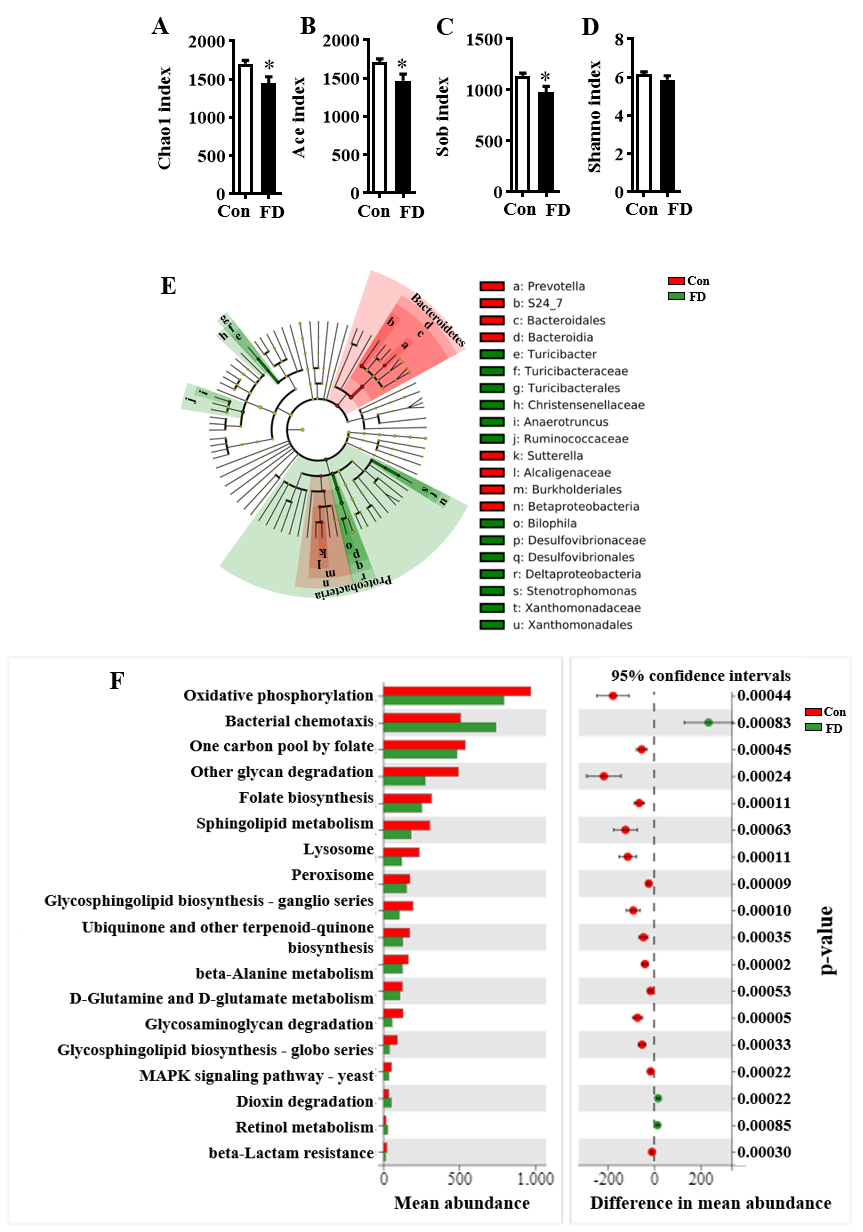


**Fig. S2. Dietary fiber deficiency altered gut microbiota.** (A-D) The α-diversity of the cecal microbiome among two groups depicted according to Chao1 index (A), Ace index (B), Sob index (C) and Shannon index (D). (E) Cladogram generated from linear discriminant analysis (LDA) (n=5-6). (F) Predicted KEGG functional pathway differences at level 3 inferred from 16S rRNA gene sequences using PICRUSt. Values are mean ± SEM. **p* < 0.05 fiber deficiency (FD) group vs. control (Con) group.

**Fig. S3**


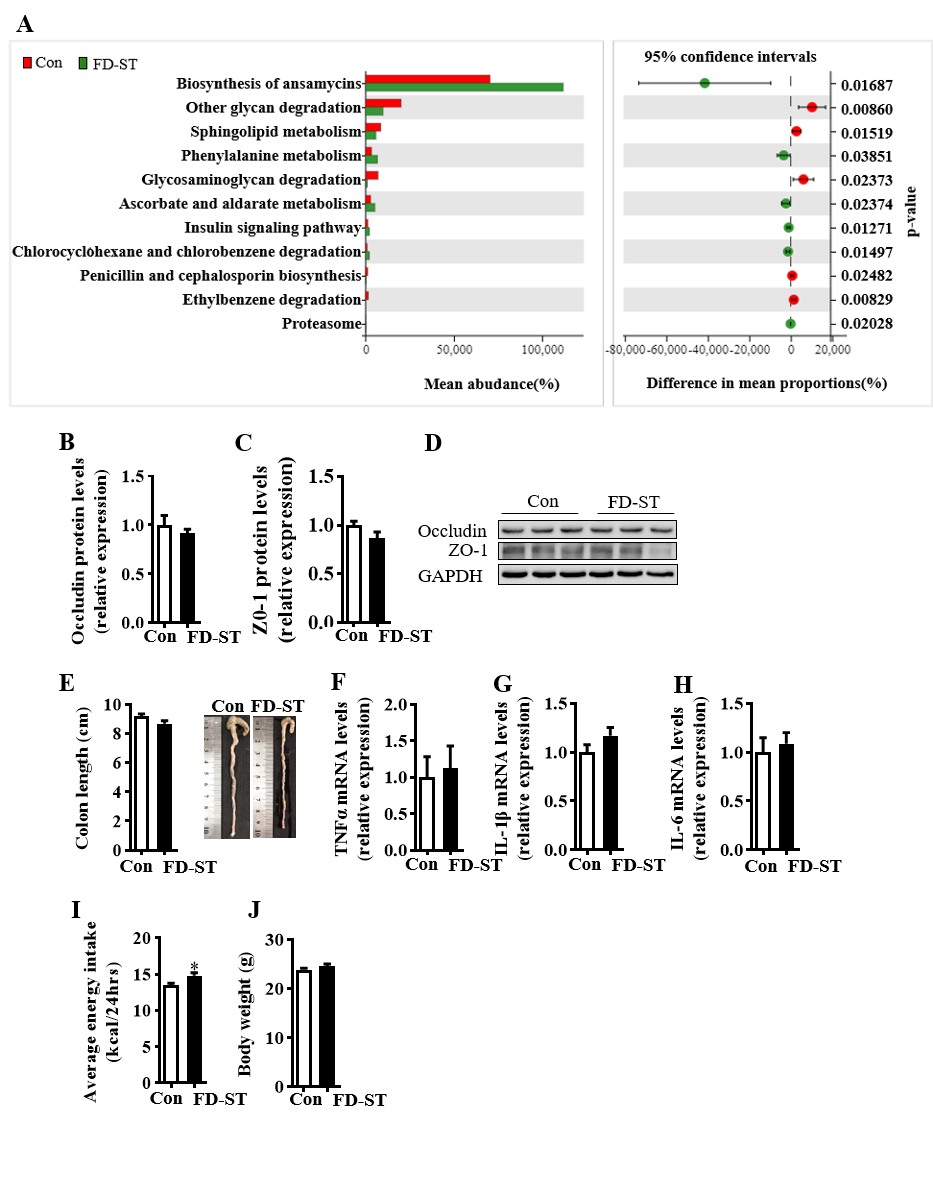


**Fig S3. Dietary fiber deficiency for 7 days altered gut microbiota, but not colon tight junctions and inflammation.** (A) Predicted KEGG functional pathway differences at level 3 inferred from 16S rRNA gene sequences using PICRUSt (n=5-6). (B-D) Protein levels of occludin and ZO-1 in the colon (n=6). (E) The quantification of colon length was statistically analyzed (n=9) with representative images of colons. (F-H) mRNA expression levels of TNFα，IL-1β and IL-6 in the colon (n=6). (I) Average energy intake (n=15). (J) Body weight at 1 week (n=15). Values are mean ± SEM. **p* < 0.05 fiber deficiency for short-term (FD-ST) group vs. control (Con) group.

**Fig. S4**


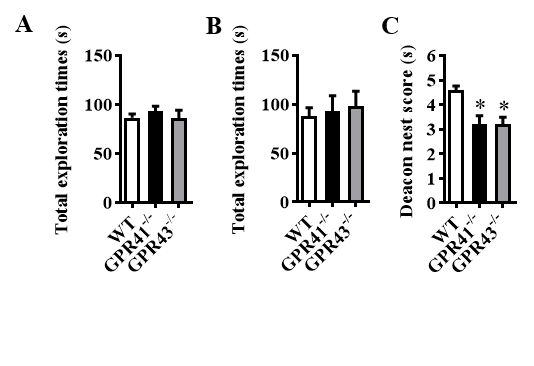


**Fig. S4 The cognitive behavior in the GPR41^-/-^ and GPR43^-/-^mice.** Total object exploration time in the object location test (A) and temporal order memory tests (B). (C) Deacon nest score in nest building tests (n=8-10). Values are mean ± SEM. **p* < 0.05 vs. wild type (WT) mice.

**Fig. S5**


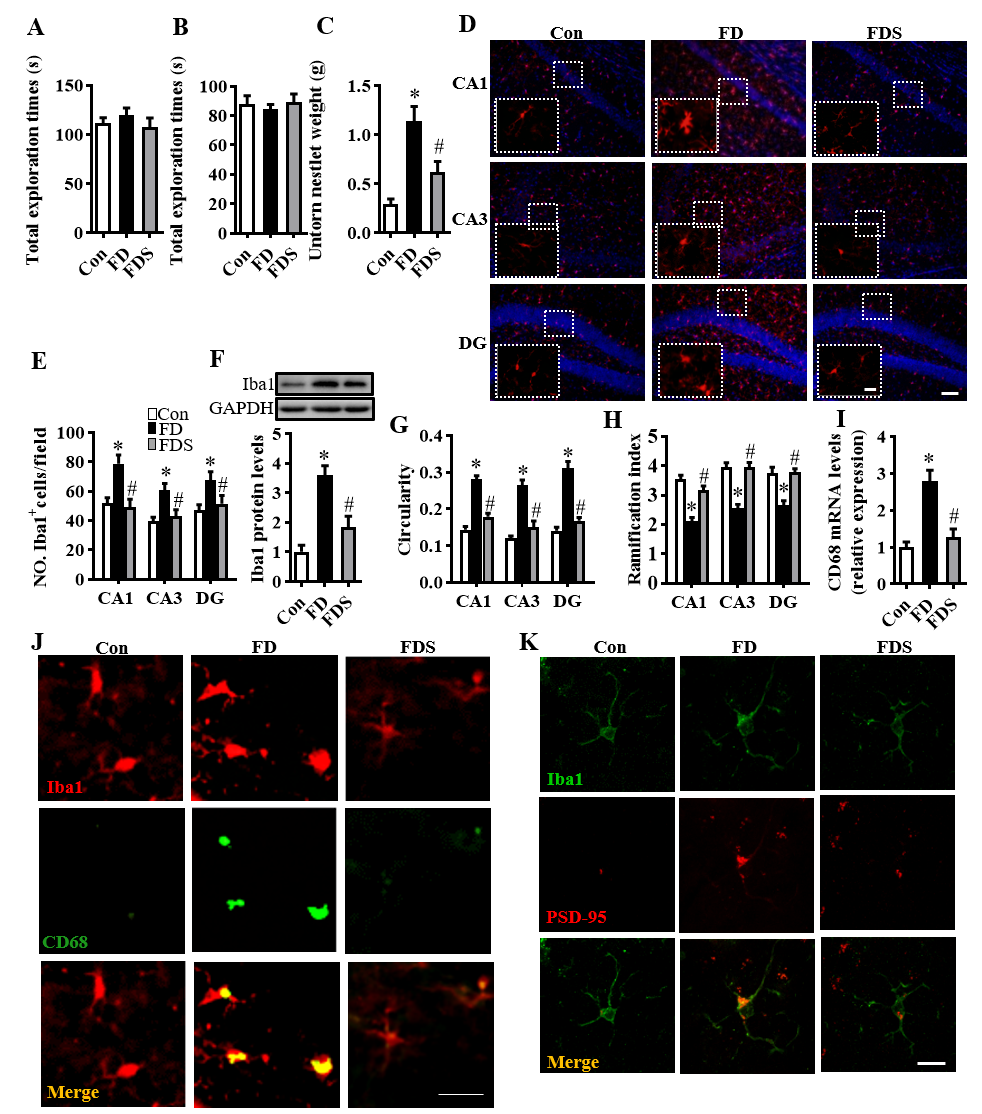


**Fig. S5. SCFAs supplementation prevented FD-induced cognitive decline and microglia activation.** Total object exploration time in the object location test (A) and temporal order memory tests (B). (C) Deacon nest score in nest building tests (n=15). (D) The immunofluorescent staining of Iba1 (Scale bar: 50μm), the image capture from the box marked with a dotted line (Scale bar: 10μm). (E) Quantification of Iba1^+^cells numbers in CA1, CA3 and DG of the hippocampus (2 images per mouse, n=6). (F) The protein level of Iba1 in the hippocampus (n=6). (G and H) The circularity and ramification index of Iba1^+^ cells (2 images per mouse, n=3). (I) mRNA expression level of CD68 (n=6). (J) The representative immunofluorescent staining of CD68 in the hippocampus, Scale bar: 25μm. (K) The orthogonal view of the high-resolution confocal image shows the colocalization of Iba1 (green) and PSD95 (red) (Scale bar: 5μm). Values are mean ± SEM. **p* < 0.05 vs. control (Con) group. ^#^*p* < 0.05 vs. fiber deficiency (FD) group. The FD mice with SCFAs supplementation: FDS.

**Fig. S6**

**
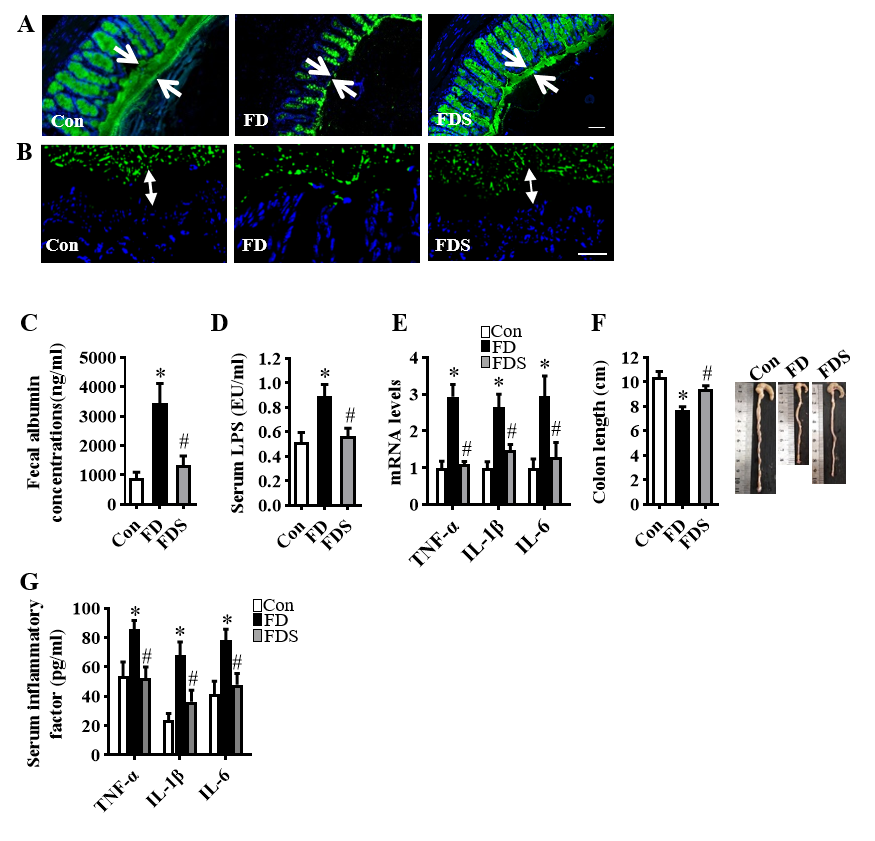
**

**Fig.S6. SCFAs supplementation prevented FD-induced intestinal integrity impairment, endotoxemia and systemic inflammation.** (A) Immunofluorescence images of colonic sections stained with Anti-MUC2 antibody and DAPI. Opposing white arrows with shafts delineates the mucus layer. Scale bar: 50μm (B) FISH analysis of sections of the colon using the general bacterial probe EUB338-Alexa Fluor 488 (green), and nuclear staining DAPI (blue). Arrows indicate the distance between bacteria and epithelium. Scale bar: 20μm. (C) Fecal albumin concentrations (n=8). (D) Serum LPS endotoxin level (n=10). (E) mRNA expression levels of TNFα, IL-1β and IL-6 in the colon (n=5). (F) The quantification of colon length was statistically analyzed (n=9) and representative images of colons. (G) TNF-α, IL-1β and IL-6 levels in the serum (n=10). Values are mean ± SEM. **p* < 0.05 vs. control (Con) group. ^#^*p* < 0.05 vs. fiber deficiency (FD) group. The FD mice with SCFAs supplementation: FDS

**Table S1**

Composition of the control (Con) and fiber deficient (FD) diets

|  |  |  | Con | |  | FD | | |
| --- | --- | --- | --- | --- | --- | --- | --- | --- |
| Energy (% kal) | |  |  | |  |  | |  |
| Fat | | | 13.1 | |  |  | | 12.2 |
| Protein | |  | 28.7 | |  |  | | 21.3 |
| Carbohydrate | |  | 58.2 | |  |  | | 66.5 |
| Fiber (% by weight) | |  | 20 | |  |  | | 5.1 |
|  | Chemical composition (%) | | | |  | Ingredient (% by weight) | | |
|  | Fat |  | 5 | |  | Soya Oil | | 5 |
|  | Nitrogen-free extract |  | 50 | |  |  |  |  |
|  | Starch |  | 29.4 | |  | Corn Starch | | 31.5 |
|  | Sucrose+glucose+fructose |  | 1.16+0.23+0.27=1.66 | |  | Sucrose | | 31.5 |
|  | Fiber |  | 20 | |  | Cellulose |  | 5.1 |
|  | Protein |  | 24.6 | |  | Casein | | 14 |
|  |  |  |  |  |  | Gelatin |  | 5 |
|  | Minerals |  | 6.1 | |  | Mineral Mix | | 5 |
|  | Various vitamins |  | 0.3-2200pm | |  | Vitamin Mix | | 1.3 |

Control (Con) diet was lab chow diet (LabDiet 5010) with 20% dietary fiber by weight from plant polysaccharide, containing high microbiota-accessible carbohydrates derived from a diverse source of plants, including corn, soybean, wheat, oats, alfalfa, and beet. Fiber deficient (FD) diet was a fiber deficiency diet with 5% dietary fiber by weight from cellulose. Simple carbohydrate or monosaccharides (sucrose and corn starch) in the FD diet replace microbiota-accessible carbohydrates in the Con diet. The FD diet was made from semi-synthetic materials according to the recommendation of “AIN93 Diet for Laboratory Rodents”.

*Chemical composition (%) of Con diet (LabDiet 5010) is calculated from the natural ingredients, including ground corn, dehulled soybean meal, wheat middlings, fish meal, whole wheat, wheat germ, brewers dried yeast, ground oats, dehydrated alfalfa meal, porcine animal fat, ground soybean hulls, calcium carbonate, dried beet pulp, salt, soybean oil, DL-methionine, pyridoxine hydrochloride, choline chloride, menadione dimethylpyrimidinol bisulfite (source of vitamin K), thiamine mononitrate, cholecalciferol, dicalcium phosphate, silicon dioxide, vitamin A acetate, folic acid, biotin, dl-alpha tocopheryl acetate, calcium pantothenate, riboflavin, nicotinic acid, vitamin B12, manganous oxide, zinc oxide, ferrous carbonate, copper sulfate, zinc sulfate, calcium iodate, cobalt carbonate.

**Table S2**

Pearson correlations between energy intake and metabolic and behavior parameters

|  | Average energy intake | |  |  |
| --- | --- | --- | --- | --- |
| Metabolic and behavior parameters | r | *P* |  |  |
| Accumulated weight gain | 0.61 | 0.016 |  |  |
| Fat weight (eWAT+ iWAT + iBAT)  Exploration time with novel place object  Exploration time with old familiar object  Deacon nest score  Untore nestlet weight | 0.79  -0.75  -0.65  -0.64  0.72 | 0.006  0.001  0.008  0.010  0.003 |  |  |

Note: eWAT: epididymal white adipose tissue; iWAT: inguinal white adipose tissue; iBAT: interscapular brown adipose tissue.

**Supplemental Methods**

**Behavioral tests**

The object location, temporal order memory, and nesting behavior tests were performed to examine dietary effects on recognition memory and spontaneous rodent behaviors. Tests were conducted similar to previous studies[1, 2]. In the object location test, the place discrimination index was calculated by using the formula: the time spent with the object moved to a novel place/the total time spent in exploring both the object moved to a novel place and the object remaining in the familiar place × 100. The temporal order memory test comprised two sample trials and one test trial with an inter-trial interval of 60 min between each trial. In each sample trial, the mice were allowed to explore two copies of the same object for 4 min; however, the objects were different between the two sample trials (sample trial 1: object A and A’; sample trial 2: object B and B’). During the test trial, one object from sample trial 1 (A; old familiar) and another object from sample trial 2 (B; recent familiar) was presented. The animals were allowed to explore the open-field for 3 min. A discrimination ratio was calculated by using the formula: [(old familiar time − recent familiar time)/total exploration time]. Intact object recognition memory for temporal order was considered if the mice spent more time exploring the old familiar object than the recent familiar object. For the nesting behavior test, the Deacon nest score and the untore nestlet weight was used to evaluate spontaneous rodent behavior (the ability of daily living). Similar to previous studies [1, 3], one hour before the dark phase, the mice were transferred into individual cages with wood-chip bedding. A nestlet pressed-cotton square (3.0 g) was put into each cage. The next morning, remaining nests were scored on a rating scale of 1-5 (1: Nestlet not noticeably touched or >90% intact, 2: Nestlet 50–90% remaining intact; 3: Nestlet 50–90% shredded, but no identifiable nest site. 4: Nestlet >90% shredded, flat nest within ¼ of the cage, 5: A (near) perfect nest with walls higher than the mouse body height for >50% of its circumference). The untore nestlet pieces were weighed. The definition of an untorn piece is more than approximately 0.1g.

**Mass-Spectrometry-Based proteomics**

***Protein Extraction:*** Mouse hippocampus was ground by liquid nitrogen into cell powder and then transferred to a 5-mL centrifuge tube. After that, four volumes of lysis buffer (8 M urea, 1% Protease Inhibitor Cocktail) was added to the cell powder, followed by sonication three times on ice using a high-intensity ultrasonic processor (Scientz). The remaining debris was removed by centrifugation at 12,000 g at 4 °C for 10 min. Finally, the supernatant was collected, and the protein concentration was determined with the BCA kit according to the manufacturer’s instructions. The aliquots were stored at -80 °C for further proteomic.

***Trypsin digestion:*** The protein reduction was performed for 30 min at 56 °C with 5 mM dithiothreitol, which was followed by alkylation with 11 mM iodoacetamide for 15 min at room temperature in darkness. The protein sample was then diluted by adding 100 mM TEAB to urea concentration less than 2M. Finally, trypsin was added at 1:50 trypsin-to-protein mass ratio for the first digestion overnight and 1:100 trypsin-to-protein mass ratio for a second 4 h digestion.

[***Tandem Mass Tag (***](https://www.thermofisher.com/au/en/home/life-science/protein-biology/protein-mass-spectrometry-analysis/protein-quantitation-mass-spectrometry/tandem-mass-tag-systems.html)***TMT) labeling:*** After trypsin digestion, the peptide was desalted by Strata X C18 SPE column (Phenomenex) and vacuum-dried. The peptide was reconstituted in 0.5 M TEAB and processed according to the manufacturer’s protocol for the TMT kit/iTRAQ kit. Briefly, one unit of TMT reagent was thawed and reconstituted in acetonitrile. The peptide mixtures were then incubated for 2 h at room temperature and pooled, desalted and dried by vacuum centrifugation.

***High-performance liquid chromatography (HPLC) fractionation*:** The sample was then fractionated by high pH reverse-phase HPLC using an Agilent 300Extend C18 column (5 μm particles, 4.6 mm ID, 250 mm in length). Briefly, the peptides were first separated with a gradient of 2% to 60% acetonitrile in 10 mM ammonium bicarbonate pH 10 over 80 min into 80 fractions. Then, the peptides were combined into 18 fractions and dried by vacuum centrifugation.

***Liquid chromatography-tandem mass spectrometry (LC-MS/MS) analysis:*** Peptides dissolved in 0.1% formic acid were directly loaded onto a reversed-phase pre-column (Acclaim PepMap 100, Thermo Scientific). Peptide separation was performed with a reversed-phase analytical column (Acclaim PepMap RSLC, Thermo Scientific). The gradient comprised of an increase from 6% to 23% solvent B (0.1% formic acid in 98% acetonitrile) over 26 min, 23% to 35% in 8 min and 35% to 80% in 3 min, then holding at 80% for the last 3 min, all at a constant flow rate of 400 nL/min on an EASY-nLC 1000 Ultra Performance Liquid Chromatography (UPLC) system (Thermo Scientific). The resulting peptides were analyzed by a Q Exactive Plus Hybrid Quadrupole-Orbitrap mass spectrometer (Thermo Scientific). The peptides were subjected to nanospray-ionization followed by tandem mass spectrometry (MS/MS) in a Q Exactive Plus (Thermo Scientific) coupled online to the UPLC. Intact peptides were detected in the Orbitrap at a resolution of 70,000. Peptides were selected for MS/MS using normalized collision energy set at 30; ion fragments were detected in the Orbitrap at a resolution of 17,500. A data-dependent procedure that alternated between one MS scan followed by 20 MS/MS scans was applied for the top 20 precursor ions above a threshold ion count of 10,000 in the MS survey scan with 30.0 s dynamic exclusion. The electrospray voltage applied was 2.0 kV. Automatic gain control was used to prevent overfilling of the Orbitrap; 50,000 ions were accumulated to generate MS/MS spectra. For MS scans, the m/z scan range was 350 to 1800. The fixed first mass was set as 100 m/z.

***Database search:*** The resulting MS/MS data were processed using the Mascot search engine (v.2.3.0). Tandem mass spectra were searched against the SwissProt human database. Trypsin/P was specified as the cleavage enzyme, allowing up to 4 missing cleavages. The mass error was set to 20 ppm for precursor ions and 0.02 Da for fragment ions. Carbamidomethylation of cysteine residues was specified as a fixed modification, and methionine oxidation was specified as a variable modification. For the protein quantification method, TMT-6-plex was selected in Mascot. The FDR was adjusted to < 1%, and the peptide ion score was set at≥40. A total of 6959 proteins were detected in all samples, in which 5823 proteins could be quantified.

***Bioinformatics for the result of proteomic:*** Gene Ontology (GO) proteome annotation was performed using the UniProt-GOA database (www. http://www.ebi.ac.uk/GOA/). First, the identified protein ID was converted to a UniProt ID and then mapped to GO IDs by protein ID. For identified proteins that were not annotated by the UniProt-GOA database, InterProScan software was used to assign GO annotation to the protein based on a protein sequence alignment method. The proteins were then classified by GO annotation based on the biological process.

In the GO enrichment analysis, proteins were classified by GO annotation into two categories: down-regulated biological process and up-regulated biological process. For each category, a two-tailed Fisher’s exact test was employed to compare the enrichment of the differentially expressed protein against all identified proteins. The Kyoto Encyclopedia of Genes and Genomes (KEGG) database was used to identify enriched pathways by a two-tailed Fisher’s exact test to compare the enrichment of the differentially expressed protein against all identified proteins. These pathways were classified into hierarchical categories according to the KEGG website (http://www.genome.jp/kegg/). In the two functional enrichment analyses (GO and KEGG pathway), a corrected p-value < 0.05 was considered significant.

All protein name identifiers were searched against the STRING database (https://stringdb.org/, v.10.5) for protein-protein interactions. Only interactions between the proteins belonging to the searched data set were selected, thereby excluding external candidates. STRING defines a metric called the confidence score to define the confidence of the interaction; we fetched all interactions that had a confidence score ≥ 0.7 (medium confidence). The interaction network formed in STRING was visualized in Cytoscape (http://www.cytoscape.org/, v.3.1). A graph-theoretical clustering algorithm and molecular complex detection (MCODE; https://omictools.com/molecular-complexdetection-tool) were used to analyze densely connected regions.

**Transmission electron microscopy (TEM)**

Mice were transcardially perfused with 4% paraformaldehyde after sacrifice. The hippocampal CA1 region was collected and rapidly fixed in a solution composed of 4% paraformaldehyde and 2.5% glutaraldehyde, following a wash with 0.1M phosphate buffer solution (PBS, pH 7.4) and postfixed with 1% osmic acid for 2 h. Subsequently, the tissue was washed with double distilled water and dehydrated with an ethanol and acetone gradient. Samples were embedded with different concentrations of epoxy resin and polymerized at 37°C for 24 h, followed by 45°C for 24 h and 60°C for 24 h. The samples were cut into ultrathin sections (70 nm) and stained with uranyl acetate and lead citrate. Two grids per specimen and 10 photographs per grid were randomly taken of the synaptic terminals and viewed on a transmission electron microscope (FEI Tecnai G2 Spirit TWIN, America) to estimate synaptic morphometry. Gray type I synapses (asymmetric synapses considered to mediate excitatory transmission) were identified in the micrographs by the presence of synaptic vesicles (SVs) and dense material in the postsynaptic axon terminal. The postsynaptic density (PSD) thickness was evaluated as the length of a perpendicular line traced from the postsynaptic membrane to the most convex part of the synaptic complex. The widths of the synaptic clefts (SCs) were estimated by measuring the widest and narrowest portions of the synapse and then averaging these values.

**Immunohistochemistry**

At a temperature of -18°C, 20µm frozen brain sections (hippocampus) were cut using a cryostat from Bregma-3.3 mm to - 4.16 mm according to a standard mouse brain atlas.^[4]^ The brain slices were blocked with 10% goat normal serum for 15 min at room temperature and then incubated with the primary antibodies at 4 °C overnight. The primary antibody anti-Iba1 (Wako, 019-19741), anti-CD68 (BIO-RAD, MCA1957T) and PSD95(CST, 3450) were used. After washing with PBS, the sections were incubated with the secondary antibodies at 37 °C for 1 h. The secondary antibody Alexa Fluor® 594 (Abcam, 150160) and Alexa Fluor® 488 (ab150117) were used. Finally, the sections were counterstained with DAPI (Sigma, D9542). The morphology of microglia (Iba1) in the CA1, CA3 and DG of the hippocampus was then imaged with a microscope (OLYMPUS IX51). Quantification of positively stained cells in the CA1, CA3, and DG regions were counted using ImageJ. In Sholl analysis for microglia morphology, images obtained from the CA1, CA3, and DG regions were thresholded and processed by a cleaning algorithm, including size-based particle exclusion and manual pruning of overlapping cell profiles. For each subsequent profile, the morphological parameters of area and perimeter were calculated. Circularity was calculated by the following formula: 4π × (area/perimeter2). The ramification index of Iba1^+^ cells was quantified by grid-cross analysis using the ImageJ 1.46r. The hippocampal CA1 area in brain tissue sections was imaged by a Leica SP8 confocal microscope system equipped with a 63x oil immersion objective (Leica, Germany) using identical light intensity and exposure settings stacks (z-step 0.1 μm). The images of contact between microglia and postsynaptic structures in identical 60x image stacks from sections double-labeled for Iba1 and PSD95 were processed by LAS X software (Leica, Germany). MUC2 in the colon was detected by staining the colonic tissue sections (5μm) with anti-MUC2 antibody (Abclonal, A14659) diluted 1:500 in TBS, and goat-anti-rabbit Alexa 488 conjugated antibody (1:1000) (Invitrogen, A32731) in TBS.

**Western blotting**

Mouse colon and hippocampus were homogenized in ice-cold RIPA lysis buffer, supplemented with complete EDTA-free protease inhibitor cocktail and PhosSTOP Phosphatase Inhibitor. The homogenate was sonicated six times for 4 sec, at 6-sec intervals on ice, and then centrifuged at 12,000 g for 20 min at 4 °C. The supernatant was collected, and the protein concentration was quantitated by BCA assay. Equal amounts of protein were separated by sodium dodecyl sulfate-polyacrylamide gel electrophoresis (SDS-PAGE) and transferred onto polyvinylidene difluoride (PVDF) membranes. The membrane was blocked with 5% non-fat milk at room temperature for 1 hr, and then incubated with the primary antibody at 4°C overnight. These primary antibodies were included: anti-Occludin (Abcam, ab167161), anti-ZO1 (Abcam, ab96587), anti-Iba1 (Wako, 019–19741), anti-p-GSK-3β(Ser9) (CST,9322S), anti-GSK-3-β (CST,12456T), anti-Tau5 (Abcam, ab80579), anti-p-Tau (S202 + T205) (Abcam, ab80579), anti-Synaptophysin (Abcam, ab32127), anti-PSD95 (CST, 3450), anti-PTP1B (Abcam, ab189179), GAPDH (ABclonal, AC033) and β-Actin (ABclonal, AC026), anti-SV2C (Thermo, PA5-68340), anti-GAP43 (CST, 8945S), anti-CAMK2d (Santa, sc-100362), anti-p-CAMKⅡ (Thr286) (CST, 12716S ), anti-CAMKⅡ (CST, 3362S). Following 3 washes in TBST, the membrane was incubated with HRP inked anti-rabbit IgG secondary antibody (CST, 7074) or HRP-linked anti-mouse IgG secondary antibody (CST, 7076S) at room temperature for 1 h. After washing 3 times with TBST, the protein bands were detected with Clarity™ ECL Western Blot substrate (Bio-Rad, 1,705,060) and visualized using the ChemiDoc Touch imaging system (Bio-Rad).

**Quantitative RT-PCR**

Total RNA was extracted from tissues homogenized in Trizol (Thermo Fisher Scientific, Waltham, MA, USA). One microgram of purified RNA was converted into cDNA with a High-Capacity cDNA Reverse Transcription Kit (Takara, Dalian, China), and the resulting cDNA was used for quantitative PCR on a real-time PCR detection system (Bio-Rad, Hercules, CA, USA). The relative mRNA expression level was determined with the 2-ΔΔCt method with GAPDH as the internal reference control. Primer sequences were as the following:

mTNFα--forward (F): CTTGTTGCCTCCTCTTTTGCTTA, mTNFα--reverse (R): CTTTATTTCTCTCAATGACCCGTAG; mIL-1β--forward (F): TGGGAAACAACAGTGGTCAGG, mIL-1β--reverse (R): CTGCTCATTCACGAAAAGGGA; mIL-6--forward (F): TCACAGAAGGAGTGGCTAAGGACC, mIL-6--reverse (R): ACGCACTAGGTTTGCCGAGTAGAT; mReg3γ--forward (F): 5’TTCCTGTCCTCCATGATCAAA-3’, mReg3γ--reverse (R): 5’CATCCACCTCTGTTGGGTTC-3; mCD68--forward (F): TCACCTTGACCTGCTCTCTCTAA, mCD68--reverse (R): GCTGGTAGGTTGATTGTCGTCTG. mGAPDH--forward (F): AGAAGGTGGTGAAGCAGGCATC, mGAPDH--reverse (R): CGAAGGTGGAAGAGTGGGAGTTG.

**Caecal microbiota (16S rRNA gene sequencing) and analysis**

Genomic DNA amplification and sequencing were conducted as in our previous study.^[5]^ Briefly, microbial DNA was extracted from the cecal contents of mice using the E.Z.N.A. stool DNA Kit (Omega Bio-tek, Norcross, GA, U.S.) according to the manufacturer’s protocols. The 16S rDNA V3-V4 region of the Eukaryotic ribosomal RNA gene was amplified by PCR (95°C for 2 min, followed by 27 cycles at 98°C for 10 sec, 62°C for 30 sec, and 68°C for 30 sec; and a final extension at 68°C for 10 min) using primers 341F: CCTACGGGNGGCWGCAG; 806R: GGACTACHVGGGTATCTAAT, where the barcode is an eight-base sequence unique to each sample. PCR reactions were performed in triplicate 50 μL mixture containing 5 μL of 10 × KOD Buffer, 5 μL of 2.5 mM dNTPs, 1.5 μL of each primer (5 μM), 1 μL of KOD Polymerase, and 100 ng of template DNA. Amplicons were extracted from 2% agarose gels and purified using the AxyPrep DNA Gel Extraction Kit (Axygen Biosciences, Union City, CA, U.S.) according to the manufacturer’s instructions and quantified using QuantiFluor-ST (Promega, U.S.). Purified amplicons were pooled in equivalent molar and paired-end sequences (2 × 250) on an Illumina platform Hiseq 2500 platform from Genedenovo Biotechnology (Guangzhou, Guangdong, China).

Raw data containing low-quality reads or chimeras would affect the reliability of subsequent analyses. To control this, we used a filter process in the raw data. Reads containing more than 10% N base and less than 60% of bases with a Q-value of >20 were deleted. The paired-end clean reads were stitched into raw tags using FLASH (v 1.2.11) with a minimum match length of 10 bp and overlap region mismatch rates of 2%. The raw tags were further filtered to obtain clean tags by using the standard Quantitative Insights into Microbial Ecology (QIIME) (V1.9.1) pipeline under specific filtering conditions. Clean tags were searched against the reference database (http://drive5.com/uchime/uchime_download.html) to perform Reference-based chimera checking using UCHIME algorithm (http://www.drive5.com/usearch/manual/uchime_algo.html). All chimeric tags were removed and finally obtained effective tags were used for further analysis.

All of the valid reads from all samples were clustered into operational taxonomic units (OTUs) based on 97% sequence similarity using UPARSE pipeline.^[6]^ The tag sequence with the highest abundance was selected as a representative sequence within each cluster. The representative sequences were classified into organisms by a naive Bayesian model using RDP classifier ^[7]^ (Version 2.2) based on Greengenes Database ^[8]^ (<https://www.arb-silva.de/>). The abundance statistics of each taxonomy were constructed in a Perl script and visualized using SVG. Linear discriminant analysis coupled with effect size (LEfSe) was performed with LEFSE software. Chao1, Shannon and all other alpha diversity indexes were calculation in QIIME. Statistics of Alpha index comparison between groups was calculated by a Welch's t-test in R software (ver. 3.1). The variation between the experimental groups (β-diversity) was assessed with principal coordinate analysis (PCoA) plots. Statistics of Welch's t-test was calculated in R software (ver. 3.1). The predicted functional composition of the intestinal microbiome was inferred for each sample using Phylogenetic Investigation of Communities by Reconstruction of Unobserved States (PICRUSt).^[9]^

**Measurement of serum cytokines, LPS and SCFAs**

ELISA kits were used to measure TNF-α, IL-6 and IL-1β of serum according to the manufacturer’s instructions (Thermo Fisher, USA). The concentration of circulating serum LPS was measured by enzyme-linked immunosorbent assay (Limulus assay kit, Cat.18110115, China). The absorbance was measured at 545 nm using a spectrophotometer, with measurable concentrations ranging from 0.1 to 1.0 EU/ml.

GC−MS analysis of short-chain fatty acid composition in the serum was performed as previously described.^[10]^ Briefly, the separation was performed on an Agilent HP-INNOWAX capillary column (30 m × 0.25 mm × 0.25 μm). The column temperature was held at 90°C for 1 min, increased to 120°C at 10°C/min, held for 8 min, then increased to 150°C at 5°C/min, before being increased to 250°C at 25°C/min, and held for 2 min. The injection volume was 1.0 μL with a split ratio 10:1. The carrier gas was high-purity helium with a flow rate of 1.0 mL/min. The mass spectrometer was operated in electron impact mode (70 eV) at 0.2 s/scan and recorded over the mass range of m/z 50−500, and a solvent delay time of 2 min. The inlet, interface, and ionization source temperatures were 250°C, 230°C, and 250°C, respectively.

**Mucus thickness measurement and bacteria localization in the colon**

Post Carnoy’s fixation, the methanol-stored colon samples were embedded in paraffin, cut into thin sections (5μm) and deposited on glass slides. Alcian blue staining was performed by the protocols as previously published.^[11]^ The thickness of the colonic sections was then measured (10 measurements per section/2 sections per animal/5 animals per group) using ImageJ after cross-validation using anti-MUC2 staining.

The staining of bacteria localization at the surface of the intestinal mucosa was conducted as previously described.^[12]^ Briefly, transverse colonic tissues full of fecal material were placed in methanol-Carnoy’s fixative solution (60% methanol, 30% chloroform, 10% glacial acetic acid) for a minimum of 3 h at room temperature. Tissues were then washed in methanol 2x 30 min, ethanol 2x 20 min, and xylene 2x 20 min and embedded in paraffin for 5 μm sections on glass slides. The tissue sections were dewaxed by preheating at 60°C for 10 min, followed by xylene 60°C for 10 min, xylene for 10 min and 100% ethanol for 10 minutes. Deparaffinized sections were incubated at 37°C overnight with EUB338 probe (5′-GCTGCCTCCCGTAGGAGT-3′) diluted to 10 μg/mL in hybridization buffer (20 mM Tris–HCl, pH 7.4, 0.9 M NaCl, 0.1% SDS, 20% formamide). After incubating with wash buffer (20 mM Tris–HCl, pH 7.4, 0.9 M NaCl) for 10 min and 3x 10 min in PBS sequentially, the tissue sections were mounted in DAPI containing mounting medium.

**References**

1. Deacon, R.M., *Assessing nest building in mice.* Nat Protoc, 2006. **1**(3): p. 1117-9.

2. Hattiangady, B., et al., *Object location and object recognition memory impairments, motivation deficits and depression in a model of Gulf War illness.* Front Behav Neurosci, 2014. **8**: p. 78.

3. Hase, Y., et al., *The effects of environmental enrichment on white matter pathology in a mouse model of chronic cerebral hypoperfusion.* Journal of cerebral blood flow and metabolism : official journal of the International Society of Cerebral Blood Flow and Metabolism, 2018. **38**(1): p. 151-165.

4. Paxinos, G. and K.B.J. Franklin, *The Mouse Brain in Stereotaxic Coordinates, 1st edn., Academic Press, San Diego.* 2002.

5. Zhang, P., et al., *Alterations to the microbiota-colon-brain axis in high-fat-diet-induced obese mice compared to diet-resistant mice.* J Nutr Biochem, 2019. **65**: p. 54-65.

6. Edgar, R.C., *UPARSE: highly accurate OTU sequences from microbial amplicon reads.* Nat Methods, 2013. **10**(10): p. 996-8.

7. Wang, Q., et al., *Naive Bayesian classifier for rapid assignment of rRNA sequences into the new bacterial taxonomy.* Appl Environ Microbiol, 2007. **73**(16): p. 5261-7.

8. DeSantis, T.Z., et al., *Greengenes, a chimera-checked 16S rRNA gene database and workbench compatible with ARB.* Appl Environ Microbiol, 2006. **72**(7): p. 5069-72.

9. Langille, M.G., et al., *Predictive functional profiling of microbial communities using 16S rRNA marker gene sequences.* Nat Biotechnol, 2013. **31**(9): p. 814-21.

10. Han, L.D., et al., *Plasma esterified and non-esterified fatty acids metabolic profiling using gas chromatography-mass spectrometry and its application in the study of diabetic mellitus and diabetic nephropathy.* Anal Chim Acta, 2011. **689**(1): p. 85-91.

11. Desai, M.S., et al., *A Dietary Fiber-Deprived Gut Microbiota Degrades the Colonic Mucus Barrier and Enhances Pathogen Susceptibility.* Cell, 2016. **167**(5): p. 1339-1353.e21.

12. Chassaing, B., R.E. Ley, and A.T. Gewirtz, *Intestinal epithelial cell toll-like receptor 5 regulates the intestinal microbiota to prevent low-grade inflammation and metabolic syndrome in mice.* Gastroenterology, 2014. **147**(6): p. 1363-77.e17.
